# Effects of harvesting of increasing intensities on genetic diversity and population structure of white spruce

**DOI:** 10.1111/eva.12064

**Published:** 2013-04-18

**Authors:** Manphool S. Fageria, Om P. Rajora

**Affiliations:** ^1^ Faculty of Forestry and Environmental Management Canadian, Genomics and Conservation Genetics Institute University of New Brunswick Fredericton NB Canada; ^2^ Present address: Agriculture and Agri‐Food Canada Fredericton NB Canada

**Keywords:** EST and genomic microsatellites, genetic diversity, harvesting of increasing intensities, *Picea glauca*, population structure, bottleneck and genetic drift, sustainable forest management, biodiversity conservation

## Abstract

Forest harvesting of increasing intensities is expected to have intensifying impacts on the genetic diversity and population structure of postharvest naturally regenerated stands by affecting the magnitude of evolutionary processes, such as genetic drift, gene flow, mating system, and selection. We have tested this hypothesis for the first time by employing widely distributed boreal white spruce (*Picea glauca*) as a model and controlled, replicated experimental harvesting and regeneration experiment at the EMEND project site (http://www.emendproject.org). We used two approaches. First, genetic diversity and population structure of postharvest natural regeneration after five harvesting treatments (green tree retention of 75%, 50%, 20%, and 10%, and clearcut) were assessed and compared with those of the unharvested control (pristine preharvest old‐growth) in two replicates each of conifer‐dominated (CD) and mixed‐wood (MW) forest, using 10 (six EST (expressed sequence tag) and four genomic) microsatellite markers. Second, genetic diversity and population structure of preharvest old‐growth were compared with those of postharvest natural regeneration after five harvesting treatments in the same treatment blocks in one replicate each of CD and MW forests. Contrary to our expectations, genetic diversity, inbreeding levels, and population genetic structure were similar between unharvested control or preharvest old‐growth and postharvest natural regeneration after five harvesting treatments, with clearcut showing no negative genetic impacts. The potential effects of genetic drift and inbreeding resulting from harvesting bottlenecks were counterbalanced by predominantly outcrossing mating system and high gene flow from the residual and/or surrounding white spruce. CD and MW forests responded similarly to harvesting of increasing intensities. Simulated data for 10, 50, and 100 microsatellite markers showed the same results as obtained empirically from 10 microsatellite markers. Similar patterns of genetic diversity and population structure were observed for EST and genomic microsatellites. In conclusion, harvesting of increasing intensities did not show any significant negative impact on genetic diversity, population structure, and evolutionary potential of white spruce in CD and MW forests. Our first of its kind of study addresses the broad central forest management question how forest harvesting and regeneration practices can best maintain genetic biodiversity and ecosystem integrity.

## Introduction

Genetic diversity is essential for adaptation, evolution, and the long‐term survival of all living organisms (Booy et al. 2000; Rajora and Mosseler 2001a). Trees are normally the keystone species of forest ecosystems, and many faunal and floral associations depend on their existence and the environment created by them. Therefore, genetic diversity of forest trees has special importance and can be viewed as the foundation of forest sustainability and ecosystem stability (Rajora and Mosseler 2001a,b). Forest trees tend to live long, so when their genetic diversity is lost, regaining it may take centuries. As such, it is crucial to conserve the genetic diversity of forest trees.

Natural and anthropogenic disturbances are major threats to the conservation of genetic diversity of forest trees. Harvesting is one of the major anthropogenic disturbances in forests that may negatively impact genetic diversity and evolutionary processes that condition genetic diversity in forest trees (e.g., Buchert et al. 1997; Rajora 1999; Rajora et al. 2000; Beaulieu et al. 2003; Finkeldey and Ziehe 2004). The evolutionary processes potentially affected by harvesting include genetic drift, gene flow, mating system, and selection (Rajora and Mosseler 2001a; Finkeldey and Ziehe 2004). The extent of genetic effects of harvesting depends upon the species and its demography, distribution, silvics and biological and ecological characteristics, and harvesting and regeneration regimes (Buchert et al. 1997; Rajora 1999; Rajora et al. 2000; Perry and Bousquet 2001; Rajora and Pluhar 2003). Negative genetic impacts include lower genetic diversity, higher inbreeding, curtailed gene flow, and increased genetic drift, which may compromise the adaptation and survival of populations.

Although harvesting in the North American temperate and boreal forests is a common practice, information is rather limited on genetic impacts of harvesting on boreal and temperate forest trees (reviewed in Krakowski and El‐Kassaby 2003). No negative genetic impacts of shelterwood, group selection (patch‐cut), and clearcut harvesting systems were observed in Douglas‐fir (*Pseudotsuga menzeisii*) (Neale 1985; Neale and Adams 1985; Adams et al. 1998) and amabilis fir (*Abies amabilis*) (El‐Kassaby et al. 2003). On the other hand, a removal of 75% trees (~seed tree cut) had significant negative impact on genetic diversity in eastern white pine (*Pinus strobus*) (Buchert et al. 1997; Rajora et al. 2000); however, no significant genetic impacts of shelterwood harvesting were detected in this species (Marquardt et al. 2007; Rajora et al., unpublished data). In western hemlock (*Tsuga heterophylla*), shelterwood harvesting caused reduction in heterozygosity (El‐Kassaby et al. 2003). Clearcut harvesting followed by natural or artificial regeneration showed no negative genetic impacts in black spruce (*Picea mariana*) (Perry and Bousquet 2001; Rajora and Pluhar 2003) and lodgepole pine (*Pinus contorta*) (Thomas et al. 1999). In contrast, post‐clearcut‐harvest plantations had significantly lower genetic diversity than unharvested old‐growth and post‐clearcut natural regeneration in white spruce *(Picea glauca*) (Rajora 1999).

Similar to the North American forests, mixed results have been reported for the genetic impacts of forest management practices in several species from Europe, Australia, and Asia (review in Finkeldey and Ziehe 2004). Forest management practices caused genetic erosion and genetic drift in Australian *Eucalyptu*s *consideniana* (Glaubitz et al. 2003a). In contrast, various regeneration practices (clear‐felling with aerial resowing and the seed tree system with site preparation) did not show significant negative effects on genetic diversity and inbreeding levels in *Eucalyptus sieberi* (Glaubitz et al. 2003b). Artificial plantations of Norway spruce (*Picea abies* Karst.) showed reduction in genetic diversity (Gömöry 1992). Ng et al. (2009) reported that selective logging impacted genetic diversity of *Shorea leprosula*, but not that of *Shorea ovalis* ssp. *Sericea* in Malaysia. Buiteveld et al. (2007) did not find significant differences in genetic diversity among 10 stands of European beech (*Fagus sylvatica*), subjected to different management regimes. Cloutier et al. (2007) found no genetic impact of selective logging on gene diversity, inbreeding, pollen dispersal, and spatial genetic structure in *Carapa guianensis* populations from Brazil. However, genetic diversity was found to be reduced and outcrossing and gene flow levels maintained after selective logging in *Hymenaea courbaril* from the same forest area in Brazil (Carneiro et al. 2011).

Forest harvesting creates population bottleneck by reducing tree density and forest cover. Bottleneck can affect genetic drift, gene flow, and mating system. Thus, higher the magnitude of bottleneck, higher effects on these evolutionary processes would be expected. Hence, it is expected that harvesting of increasing intensities would create proportionally higher negative genetic impacts in the postharvest residual and regenerated populations. However, this hypothesis remains untested because it requires long‐term controlled and replicated experimental harvesting and regeneration experiments, which are not only time‐consuming but also very expensive. Except for two studies (Adams et al. 1998; El‐Kassaby et al. 2003), all other studies are based on existing operational harvesting treatments. Adams et al. (1998) examined the effects of shelterwood, group selection, and clearcut harvesting in Douglas‐fir in a replicated experiment. The treatments involved green retention of about 67%, 33%, and 0% (clearcut) Douglas‐fir trees. However, natural regeneration could not be studied in group selection cut, and most of the harvested blocks were planted. El‐Kassaby et al. (2003) conducted their study as a part of the partially replicated MASS (Montane Alternative Silvicultural Systems) project involving shelterwood, patch‐cut, and clearcut harvesting systems. These studies involved operationally used harvesting systems and did not cover a range of harvesting intensities and postharvest natural regeneration regimes. All reported studies reviewed above examined genetic effects of shelterwood (~25% trees removal in first harvesting and another ~ 25% in second harvesting), patch‐cut (~50% tree removal), seed tree harvesting (~75% trees removal), and/or clearcut (~100% trees removal). However, a comparison of genetic effects of harvesting of ~25% to ~100% trees has not been made in the same species at the same time in one experiment at the same site. Also, all of the studies so far have used only one approach either (i) investigating genetic diversity and population structure of existing adjacent or nearby unharvested and postharvest populations or (ii) examining genetic diversity and population structure of preharvest and postharvest populations from the same treatment area. However, no study has so far used and compared both of these approaches.

The Ecosystem Management Emulating Natural Disturbance (EMEND) project (http://www.emendproject.org ), of which, one of us (O.P.R.) is a founding member, provides an ideal and unique experimental design to examine genetic effects of increasing harvesting intensities in forest trees using both approaches. The details of the experimental design are provided in Materials and Methods below. The EMEND project uses the multidisciplinary ecosystem approach to address the central forest management question how forest harvesting and regeneration practices can best maintain biodiversity, forest structure and function, and ecosystem integrity of conifer‐dominated (CD) and mixed‐wood (MW) landscapes that have originated from wildfires and other inherent natural disturbances (http://www.emendproject.org). The main areas of research are biodiversity of various organisms, genetic diversity of trees, forest productivity, forest ecosystem processes and patterns, silvicultural systems, forest fire ecology, forest soils and nutrient cycling, forest hydrology and microclimate, and socio‐economics. Therefore, a variety of research has been and is being conducted on a single land base so that it could be integrated to address a very broad forest management question about biodiversity conservation and sustainable ecosystem management. Because genetic diversity is the basis of all biodiversity and foundation of ecosystem stability, our study contributes significantly to the broad goal of the EMEND project. White spruce and trembling aspen (*Populus tremuloides*) are the dominant species in the EMEND project forest. We have chosen white spruce as a model species for our study. White spruce occurs in CD and MW forest types at the EMEND project site and in the rest of North America.

White spruce is a widely distributed transcontinental tree species of the boreal forest in Canada (Hosie 1979), where it is one of the dominant species. It is one of the most important trees for the production of wood pulp and lumber in Canada and is also an ecologically important species of the North American boreal forest ecosystem. White spruce has high genetic diversity and is a predominantly outcrossing species (e.g., Rajora et al. 2005; O'Connell et al. 2006). Although white spruce is managed under both artificial and natural regeneration systems, its artificial regeneration after clearcut harvesting is the primary practice used in North America.

We hypothesized that the genetic diversity of postharvest natural regeneration of white spruce will reduce with harvesting of increasing intensities. Lower genetic diversity with increasing harvesting intensities was expected as a result of potentially higher genetic bottleneck, reduced tree density, and increased inbreeding and genetic drift. We also expected higher negative genetic impact of harvesting of increased intensities on white spruce in MW stands than in CD stands because of lower numbers of white spruce trees per unit area, uneven stand structure, and higher barriers for white spruce pollen gene flow in MW than in CD stands. So far, there is no published information on the comparison of genetic effects of harvesting of a species from the CD and MW forests.

This study was undertaken to address the following questions: (i) Does harvesting of increased intensities result in proportional reduction in genetic diversity levels and increased inbreeding levels, and altered population genetic structure of the postharvest natural regeneration of white spruce? (ii) Does white spruce in CD and MW stands respond similarly to harvesting of increased intensities? We have used two approaches to address these questions. First, we have examined the genetic diversity and population structure of unharvested old‐growth control and the postharvest young natural regeneration of white spruce after harvesting of five intensities in two replicates each of the CD and MW forest, using 10 (six EST (expressed sequence tag) and four genomic) microsatellite markers. Second, we have assessed the genetic diversity and population structure of the preharvest old‐growth and postharvest young natural regeneration of white spruce in the same treatment blocks in one replicate each of CD and MW forests.

## Materials and methods

### Study site, experimental design, and sampling

**Figure 1:**
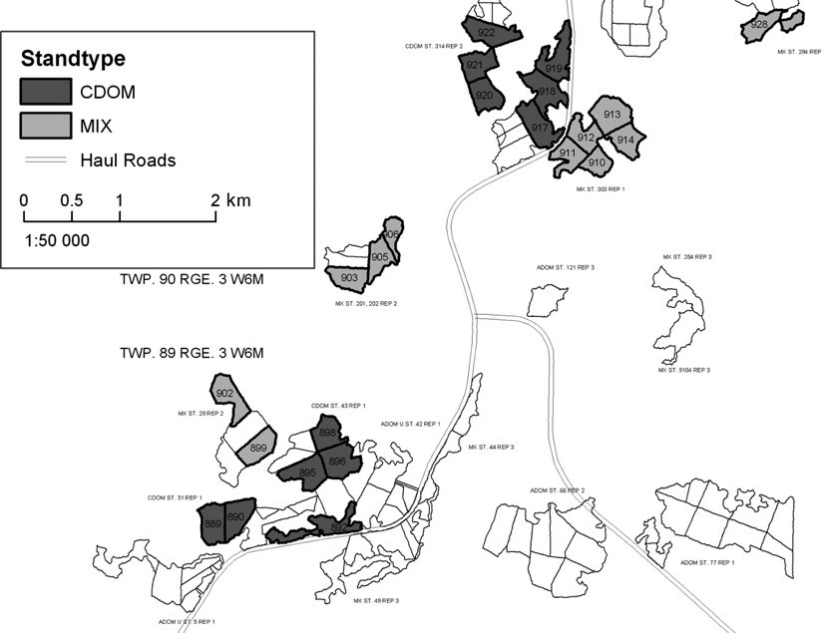
Location of the sampled subpopulations at the EMEND experimental site (Source: EMEND, 2010 http://www.emendproject.org). Details of harvesting treatments and subpopulation (block) numbers are provided in Table 1.

The EMEND (http://www.emendproject.org ) project experimental site was used for this study. The EMEND site is located in the Upper Boreal‐Cordilleran Ecoregion in the boreal forest in northern Alberta, approximately 90 km northwest of Peace River (approximate coordinates for the project center: latitude 56^o^46′13″N; longitude 118^o^22′28″W). The experiment was established in 1997 and is expected to run for 80–100 years (one stand rotation). The EMEND experimental design consists of four forest types (pristine CD, MWs, aspen‐dominated, and aspen‐dominated with spruce understory) with three replicates within each forest types, and five harvesting treatments (10% residual, 10R; 20% residual, 20R; 50% residual, 50R; 75% residual, 75R; and clearcut, CCT) within each replicate. Additionally, it followed a compact blocking system, where populations under different harvesting treatments and in the four forest types were expected to grow in basically uniform conditions. Each of the treatment blocks is approximately 10 ha in size. Harvesting was carried out in the winter of 1998. Blocks were harvested in a modified uniform shelterwood pattern so that a recommended amount of original basal area could be preserved. The feller‐buncher and wheeled skidder were used to harvest the compartments (see www.emendproject.org for a complete description). In brief, harvest operations were accomplished in 5‐m‐wide machine corridors. An interval of 20 m was maintained between machine corridors, which left a 15‐m‐wide green tree retention strip between each corridor. The retention levels, 10%, 20%, 50%, and 75%, were accomplished by systematic tree removal from the retention strips. Trees in the clearcut treatment (2% retention) were harvested in the conventional manner instead of in the designated machine corridors. Our white spruce study focused on two replicates in each of the CD and MW stands (Fig. 1). The CD stands had >70% white spruce, whereas the MW stands had 40–50% white spruce as a stand component. All of the sampled subpopulations were located within 10 km of each other (Fig. 1).

Genetic diversity and population structure of 16 preharvest pristine old‐growth subpopulations from the CD and MW forest types at the EMEND project site were previously determined by Rajora et al. (2005). Our present study was carried out to determine the impacts of harvesting of five intensities on the genetic diversity and population structure of white spruce. We used two approaches.

#### Adjacent preharvest control and postharvest natural regeneration

Genetic diversity and population structure were assessed for the unharvested old‐growth control subpopulations and naturally regenerated seedlings after five harvesting treatments (green tree retention of 75%, 50%, 20%, 10%, and clearcut with 2% tree retention) in two replicates (R1 and R2) each of the CD and MW forest types (Table 1). Therefore, a total of 24 subpopulations [2 forest types × 2 replicates × (5 harvesting treatments + 1 control)] were studied. In 2007, 35 old‐growth or postharvest naturally regenerated individuals per population were randomly sampled, separated by a minimum distance of 35 m. Four subpopulations had <35 individuals as we could not find sufficient postharvest naturally regenerated seedlings in those treatment blocks (Table 1). In these blocks, white spruce natural regeneration was poor. Needle tissues were collected from young seedlings growing in postharvest naturally regenerated blocks and mature individuals in the unharvested control. We sampled only those seedlings that were 4 years old or younger to make sure that the sampled white spruce individuals were of post‐1998 harvest origin. Insufficient number of seedlings were found in the 10% retention treatment of replicate 2 of the MW forest type, and this block could not be included in the study. In total, 780 individuals from 23 subpopulations were used in this approach (Table 1).

**Table 1:** Forest types, harvesting treatments, and white spruce subpopulations sampled

Forest type (FT)	Harvesting treatments (HT)		Rep 1 Block/subpopulation	No. samples	Rep 2 Block/subpopulation	No. samples
	Treatment	Code				
Conifer‐dominated	Unharvested control	CON	E889	35	E918	35
	75% retention	75R	E890	30	E921	35
	50% retention	50R	E898	28	E920	35
	20% retention	20R	E896	32	E919	35
	10% retention	10R	E895	25	E917	35
	Clearcut	CCT	E892	35	E922	35
Mixed‐wood	Unharvested control	CON	E928	35	E902	35
	75% retention	75R	E912	35	E906	35
	50% retention	50R	E911	35	E903	35
	20% retention	20R	E910	35	E905	35
	10% retention	10R	E913	35	No individual sampled	
	Clearcut	CCT	E914	35	E899	35

#### Genetic diversity of preharvest and postharvest subpopulations in the same blocks

In this approach, genetic diversity and population structure of 10 preharvest old‐growth subpopulations and 10 postharvest naturally regenerated seedling subpopulations from the same 10 treatment blocks (CD: E917, 919, 920, 921, 922; MW: E913, 910, 911, 912, 914; see Table 1) were assessed. For this purpose, the genotype data for 350 individuals sampled from the preharvest old‐growth subpopulations were taken from Rajora et al. (2005) and for 350 postharvest naturally regenerated seedlings sampled from the same blocks from Approach 1 above. The genotype data for only four genomic microsatellite loci (SPAG003, UAPgGT8, UAPgCA91, and PGL14) could be used as only these loci were common for the preharvest tree (Rajora et al. 2005) and postharvest seedling (Approach 1 above) genotypes.

### Microsatellite genotyping

Genomic DNA from individual 780 white spruce samples was isolated using the Qiagen MagAttract Plant DNA Extraction Kit (Qiagen, Toronto, ON, Canada) and high‐throughput magnetic fishing protocol (Bashalkhanov and Rajora 2008). Ten microsatellite markers of the nuclear genome were used for genotyping 780 individuals (Table 2). Of these, six were EST and four genomic microsatellites (Table 2). These 10 microsatellite markers were chosen for their high resolution and unambiguous allele identification after screening many genomic and EST microsatellites.

**Table 2:** Microsatellite DNA loci used and the number and size of alleles detected at each microsatellite DNA locus

Microsatellite locus^a^	Total no of alleles	Allele size range (bp)
EST		
RPGSE2	10	167–194
RPGSE5	3	242–260
RPGSE17	4	154–163
RPGSE34	29	225–281
RPGSE35	13	147–173
RPGSE44	10	200–220
Mean no of alleles	11.5	
Genomic		
SPAG003	23	106–150
PGL14	33	104–190
UAPgGT8	23	192–240
UAPgCA91	48	112–230
Mean no of alleles	31.75	
Overall mean no. of alleles	19.6	

a RPGSE2, RPGSE5, RPGSE17, RPGSE34, RPGSE35, and RPGSE44 were developed in the Rajora laboratory at Dalhousie University (details to be published elsewhere); SPAG003 is from Norway spruce (Rajora et al. 2005); PGL14 is from Rajora et al. (2001); UAPgGT8 and UAPgCA91 are from Hodgetts et al. (2001).

One primer in each pair carried a standard M13 tail sequence (forward: 5′‐CACGACGTTGTAAAACGAC‐3′; reverse: 5′‐GGATAACAATTTCACACAGG‐3′) to facilitate fluorescent labeling and detection. Amplification reactions were performed in a 10‐μL reaction volume containing 5–10 ng of template genomic DNA, 0.2 mm dNTP, 1.2–3.0 mm MgCl_2_, 0.2–1.0 pmol each primer, 0.4–1.0 pmol of fluorescent‐labeled M13 primer (5′‐IRDye 700/800), 1 ×  GoTaq flexi clear reaction buffer, and 0.25 units of GoTaq Flexi DNA Polymerase (Promega, Madison, WI, USA).

The PCR conditions to amplify the microsatellite loci RPGSE2, RPGSE17, and RPGSE44 were as follows: 95°C for 2 min followed by 30 cycles of 95°C for 20 s, 56°C for 20 s, 72°C for 30 s, and final extension at 72°C for 3 min. The PCR conditions to amplify the microsatellite loci RPGSE5, RPGSE 34, and RPGSE35 were as follows: denaturation at 94°C for 2 min followed by 35 cycles of 94°C for 20 s, 56°C for 20 s, 72°C for 1 min, and final extension at 72°C for 4 min. For SPAG003, touchdown protocol was used (Rajora et al. 2001). For the microsatellite locus PGL14, amplification was performed according to Rajora et al. (2005) with a minor modification: initial denaturation at 94°C for 3 min, followed by two cycles of 30 s each at 94°C, 60°C, and 72°C; and 38 cycles of 15 s each at 94°C, 50°C, and 72°C, followed by a final extension step for 4 min. The amplification conditions used in Rajora et al. (2005) were as follows: initial denaturation at 94°C for 3 min, followed by two cycles of 30 s each at 94°C, 60°C, and 72°C; 11 cycles of 15 s at 94°C, 60°C, and 72°C, with gradual lowering of the annealing temperature in steps from 60°C to 54°C by the eleventh cycle; and 25 cycles with 15 s each at 94°C, 54°C, and 72°C followed by a final extension step for 3 min. We have to make a minor modification to adjust for the different thermocyclers used in this (epGradients Master Cycles, Eppendorf, Germany) and Rajora et al. (2005) (PTC 200, MJ Research, Inc., Bruno, QC, Canada) studies to make sure that the genotyping results were the same for the same individuals from the two studies. The step‐down protocol (Rajora et al. 2005) was used to amplify microsatellite loci UAPgGT8 and UAPgCA91. The PCR conditions were as follows: 94°C for 3 min, 94°C for 30 s, annealing at 58°C for 30 s followed by a 10‐step touchdown decreasing by 1°C at each step, and an extension step at 72°C for 30 s. Conditions for the final 10 cycles were 30 s at 94°C, 56°C, and 72°C.

Amplification products with incorporated fluorescent labels were separated on a LI‐COR 4200 or 4300 genetic analyzer (LI‐COR, Inc., Lincoln, NE, USA). Genotypes of the sampled individuals were first scored by using SAGA GT/MX software and then verified manually.

### Data analysis

Data quality for microsatellite loci was checked with the MICROCHECKER program (van Oosterhout et al. 2004). This program provides information about the possible occurrence of null alleles if there is an overall significant excess of homozygotes and if it is evenly distributed across the homozygote classes. Standard genetic diversity parameters (total, mean, and effective number of alleles, observed and expected heterozygosity) and fixation index for individual subpopulations were calculated using the GENALEX 6 Program (Peakall and Smouse 2006). In order to determine genetic differentiation and structure of subpopulations, Nei's unbiased genetic distances (Nei 1978), Cavalli‐Sforza and Edwards' chord distances *D*_C_ (Cavalli‐Sforza and Edwards 1967), and Wright's *F*‐statistics were calculated for all subpopulations using GENALEX 6. Nei's (1978) genetic distances assume that genetic differences in populations occur due to mutation and genetic drift effects, whereas the chord distances (Cavalli‐Sforza and Edwards 1967) take only the effect of genetic drift into consideration. Harvesting treatments may have impacted genetic drift through population‐size bottlenecks. Wright's *F*‐statistics was also calculated separately for the preharvest old‐growth and postharvest seedling populations to determine whether the harvesting treatments had any effect on genetic differentiation. In order to assess deviation of *F*_IS_ from zero, permutation tests were conducted by permuting the alleles within samples over all loci in each population using the FSTAT program (Gaudet 1995). Allelic richness was also estimated using this program. Tests for allelic heterogeneity among subpopulations for 10 loci were performed using Fisher's exact test after 10 000 dememorization steps and a total of 500 000 iterations (100 batches with 5000 iterations/batch) using the GENPOP program (Raymond and Rousset 1995). To examine whether genetic distances correlate with geographical distances, analysis of isolation by distance (IBD) was performed using the Mantel test by regressing pairwise *F*_*ST*_ with pairwise geographical distances (Rousset 1997) between populations. A significance test of the regression was performed with 1000 bootstraps.

The neighbor‐joining trees from 10 microsatellite loci with 1000 bootstrap iterations were constructed based on both Nei's (1978) and chord (Cavalli‐Sforza and Edwards 1967) genetic distances using the Phylip Software Package (Felsenstein 2004). Analysis of variance (anova) and Duncan's multiple range test (DMRT) were performed to test the significance of differences in genetic diversity parameters and fixation index due to forest types and harvesting treatments, and between pre‐ and postharvest populations using SAS 9.1 software package (SAS Institute Inc 2001). The anova model used was as follows:

$$Y_{\mathrm{ijk}}=\mu +F_{i}+H_{j}+e_{\mathrm{ijk}},$$

where *Y*_*ijk*_ is the dependent genetic diversity measure of a population *k* of *j*th harvesting treatment of *i*th forest type, *μ* is the overall mean, *F*_*i*_ is the effect of the *i*th forest type (*i* = 1, 2), *H*_*j*_ is the effect of the *j*th harvesting treatment (*j* = 1, 2, 3, 4, 5, 6), and *e*_*ijk*_ is the random error component.

All of the above data analyses were performed for all 10 microsatellite loci and separately for six EST and four genomic microsatellite loci to check whether the patterns of the results were the same for the EST and genomic microsatellites.

## Effect of the number of markers

To confirm whether 10 microsatellite loci are sufficient to determine genetic diversity levels, simulated data sets for microsatellite genotypes were created by using the Markov chain‐based simulation algorithm applied in EASYPOP 2.1 (Balloux 2001). Initially, we created two large natural populations (*n* = 10 000) with 100 freely recombining loci. These populations followed an island model of migration. The parameters set to create these populations are as follows: migration rate 0.1; selfing rate 0.05; mutation rate (2 × 10^−4^) with K‐allele mutation model; all loci with 50 possible allelic states. These populations were permitted to evolve under the above‐listed evolutionary setups for 20 000 generations. Of the two populations, one population was selected for the random subsampling of 23 populations with 35 individuals for each of 100, 50, and 10 microsatellite loci. Number of alleles per locus, observed heterozygosity, expected heterozygosity, and inbreeding coefficient were calculated for each data set using the FSTAT program (Gaudet 1995). The parameters' values were averaged over the five replications in each simulated data sets and were plotted.

## Results

### Approach 1. Genetic diversity and population structure of adjacent preharvest control and postharvest natural regeneration in CD and MW stands

#### Genetic diversity

The number of alleles ranged from 3 to 29, with a mean of 11.5 alleles per locus for EST microsatellite loci, whereas the number of alleles ranged from 23 to 48, with a mean of 31.75 alleles per locus for the genomic microsatellite loci (Table 2). The results on genetic diversity parameters and fixation index (*F*) for individual preharvest and postharvest subpopulations for the CD forest are provided in Table S1 and those for the MW forest in Table S2. The means of allelic genetic diversity, heterozygosity, and *F* for different harvesting treatments in the CD and MW forest types are provided in Table 3. Overall means of genetic diversity parameters and *F* for preharvest control and postharvest natural regeneration after five harvesting treatments are in Table 4.

**Table 3:** Mean genetic diversity parameters and fixation index (*F*) for unharvested control and postharvest natural regeneration of white spruce in the conifer‐dominated (CD) and mixed‐wood (MW) forest based on 10 microsatellite loci

HT	*A*_T_	*A*	*A*_e_	*A*_R_	*A*_P_	*H*_o_	*H*_e_	*F*
CD								
CON	112.0	11.20	6.50	10.66	0.5	0.492	0.639	0.175
75R	114.5	11.45	7.15	10.38	0.5	0.529	0.656	0.191
50R	108.5	10.85	6.87	9.90	0.5	0.572	0.675	0.152
20R	108.5	10.85	6.14	9.84	0.5	0.523	0.639	0.161
10R	109.5	10.95	7.12	10.28	1.0	0.557	0.659	0.162
CCT	112.5	11.25	6.49	10.05	1.5	0.526	0.666	0.197
MW								
CON	114.0	11.40	6.21	10.24	1.0	0.537	0.656	0.171
75R	115.5	11.55	7.28	10.66	0.5	0.536	0.669	0.208
50R	114.5	11.45	6.90	10.40	0.5	0.545	0.651	0.140
20R	111.0	11.10	6.74	10.44	0.0	0.523	0.648	0.202
10R	109.0	10.90	6.82	9.71	1.0	0.440	0.645	0.253
CCT	116.5	11.65	7.03	9.84	0.0	0.553	0.658	0.127
Mean CD	110.9	11.09	6.71	10.20	0.7	0.533	0.655	0.173
Mean MW	113.8	11.38	6.83	10.30	0.5	0.529	0.655	0.177

Details of harvesting treatments (HT) are provided in Table 1. *A*_T_, total number of alleles; *A*, mean number of alleles per locus; *A*_e_, effective number of alleles per locus; *A*_R_, allelic richness; *A*_P_, private alleles; *H*_o_, mean observed heterozygosity; *H*_e_, mean expected heterozygosity. anova did not show any significant differences among harvesting treatments for all eight parameters (Table S4).

**Table 4:** Overall means of genetic diversity parameters and fixation index (*F*) and their (SE) for unharvested control and postharvest natural regeneration of white spruce in the conifer‐dominated and mixed‐wood forest based on 10 microsatellite loci

Harvesting treatment	*N*	*A*_T_	*A*	*A*_e_	*A*_R_	*A*_P_	*H*_o_	*H*_e_	*F*
CON	35	113.0	11.30 (1.118)	6.35 (0.818)	10.44	0.75	0.514 (0.036)	0.647 (0.046)	0.172 (0.028)
75R	34	115.0	11.50 (1.223)	7.21 (0.905)	10.52	0.50	0.532 (0.042)	0.662 (0.047)	0.199 (0.029)
50R	33	111.5	11.15 (1.218)	6.89 (0.861)	10.15	0.50	0.558 (0.041)	0.662 (0.046)	0.146 (0.024)
20R	34	109.8	10.98 (1.171)	6.44 (0.825)	10.14	0.25	0.523 (0.040)	0.643 (0.046)	0.181 (0.028)
10R	32	109.3	10.93 (1.360)	7.02 (1.051)	10.09	1.00	0.518 (0.045)	0.654 (0.054)	0.192 (0.039)
CCT	35	114.5	11.45 (2.596)	6.76 (1.710)	9.95	0.75	0.539 (0.079)	0.661 (0.096)	0.162 (0.051)

Details of the harvesting treatments are provided in Table 1. *N*, number of samples; *A*_T_, total number of alleles; *A*, mean number of alleles per locus; *A*_e_, effective number of alleles per locus; *A*_R_, allelic richness; *A*_P_, private alleles; *H*_o_, mean observed heterozygosity; *H*_e_, mean expected heterozygosity; anova showed no significant differences among harvesting treatments for all eight parameters (Table S4).

All 23 white spruce subpopulations showed high microsatellite genetic diversity (Tables S1 and S2). As expected, genetic diversity was higher for genomic than for EST microsatellites (Tables S5–S7 and S10–S12). Although there was some variation in allelic genetic diversity measures of subpopulations between replicates and within and between the CD and MW forests, overall genetic diversity was similar among 23 populations. The variation in the genetic diversity measures among subpopulations was not related to the harvesting treatments. For example, the preharvest old‐growth control subpopulation had the highest allelic diversity in R2 of the MW forest and the lowest in R1 of the same forest type (Table S2). Unharvested old‐growth subpopulations and postharvest natural regeneration (seedlings) after five harvesting treatments showed similar levels of genetic diversity (Tables 3 and 4), with no significant differences in genetic diversity and *F* levels between the unharvested control and postharvest natural regeneration (Table S3).

A similar level of genetic diversity was recorded for CD and MW stands in the preharvest control as well as postharvest natural regeneration (Table 3; Tables S1 and S2). All of the genetic diversity measures such as mean number of alleles (CD 11.09; MW 11.38), effective number of alleles (CD 6.71; MW 6.83), observed heterozygosity (CD 0.533; MW 0.529), and expected heterozygosity (CD 0.655; MW 0.655) were not significantly different (*P *>* *0.05) between the CD and MW stands (Table S3).

We also analyzed data for all 23 subpopulations separately for six EST microsatellite and four genomic microsatellite loci. Although EST microsatellites showed lower levels of allelic diversity (Tables S5–S7) than genomic microsatellites (Tables S10–S12), the patterns for genetic diversity and *F* for unharvested control and postharvest natural regeneration were similar to those observed from all 10 microsatellite loci (Tables 3 and 4; Supplementary Tables S1–S3). For EST microsatellites, the differences between harvesting treatments were not significant for seven measures (*P* > 0.05), but were significant for effective number of alleles (*P* = 0.038). However, the differences for *A*_e_ were not related to a specific harvesting treatment (Table S7); the most contrasting treatments (clearcut and unharvested old‐growth) had nonsignificant differences. All genetic diversity measures and *F* from genomic microsatellites were not significantly different between harvesting treatments (*P* > 0.05).

#### Population genetic structure

For all 10 microsatellite loci, the mean *F*‐statistics parameters (*F*_IS_, *F*_IT_, and *F*_ST_) were similar among unharvested control and postharvest naturally regenerated subpopulations after harvesting of different intensities (Table 5). The mean *F*_IS_ ranged from 0.146 to 0.199, with an overall mean of 0.175 for all 23 subpopulations (Table 5). All of the *F*_IS_ estimates were significantly different from 0 (*P *<* *0.05), revealing a departure from Hardy–Weinberg equilibrium with deficiency of heterozygotes. The *F*_ST_ estimates for harvesting treatments ranged from 0.020 for 75R to 0.028 for 20R, with an overall mean *F*_ST_ of 0.032 among all 23 subpopulations (Table 5). The indirect estimates of gene flow estimated from the *F*_ST_ values were high (Table 5). There was significant heterogeneity in the single‐locus *F*_IS_, *F*_IT,_ and *F*_ST_ estimates, with RPGSE17 showing the lowest and RPGSE35 the highest values (Table S4).

**Table 5:** Mean *F*‐statistic estimates (SE) and indirect gene flow rates (*N*_M_), calculated for all subpopulations combined and separately by harvesting treatments based on 10 microsatellite loci

Harvesting treatments	No. of stands	*F*_IS_	*F*_IT_	*F*_ST_	*N*_M_
All subpopulations	23	0.175 (0.041)	0.201 (0.043)	0.032 (0.008)	7.56
CON	4	0.172 (0.049)	0.191 (0.048)	0.027 (0.004)	9.01
75R	4	0.199 (0.042)	0.215 (0.043)	0.020 (0.004)	12.25
50R	4	0.146 (0.037)	0.169 (0.037)	0.026 (0.006)	9.37
20R	4	0.181 (0.045)	0.207 (0.047)	0.028 (0.007)	8.68
10R	3	0.192 (0.049)	0.209 (0.045)	0.022 (0.004)	11.11
CCT	4	0.162 (0.054)	0.187 (0.057)	0.027 (0.010)	9.01

Details of the harvesting treatments are provided in Table 1. Values in parentheses are standard error. *N*_M_: indirect migration rates calculated from *F*_ST_ estimates as *F*_ST_ = 1/4N_M_ + 1 (Crow and Aoki 1984).

Genetic distances (Nei 1978) were low among the studied subpopulations (Table 6). The lowest mean genetic distance (0.031) was observed between the 75R and 10R and the highest (0.066) between the 50R and 20R subpopulations. On average, the lowest (0.028) genetic distance was observed among subpopulations within 75R and the highest (0.055) among subpopulations within CCT (Table 6). Similar patterns of chord genetic distances were observed (data not shown).

**Table 6:** Mean and range of genetic distances (Nei 1978) among (below the diagonal) and within (at the diagonal) populations under different harvesting treatments based on 10 microsatellite loci

Harvesting treatment	No. of subpopulations	CON	75R	50R	20R	10R	CCT
CON	4	0.042 **(0.006)** (0.026–0.062)					
75R	4	0.039 (0.019–0.073)	0.028 **(0.005)** (0.016–0.042)				
50R	4	0.053 (0.020–0.116)	0.033 (0.015–0.070)	0.045 **(0.008)** (0.031–0.070)			
20R	4	0.041 (0.020–0.073)	0.046 (0.015–0.138)	0.066 (0.026–0.171)	0.050 **(0.010)** (0.016–0.086)		
10R	3	0.038 (0.025–0.059)	0.031 (0.009–0.076)	0.046 (0.000–0.103)	0.046 (0.017–0.093)	0.037 **(0.005)** (0.025–0.044)	
CCT	4	0.042 (0.021–0.082)	0.038 (0.011–0.081)	0.051 (0.021–0.121)	0.052 (0.015–0.138)	0.042 (0.021–0.105)	0.055 **(0.013)** (0.022–0.089)

Details of the harvesting treatments are provided in Table 1. Values in bold parentheses are standard errors.

**Figure 2:**
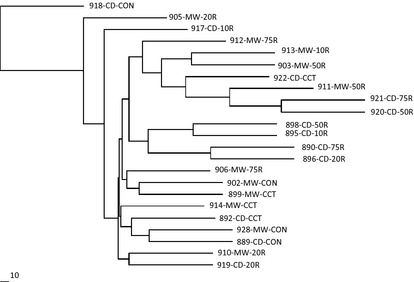
A neighbor‐joining tree, based on genetic distances (Nei 1978) from 10 microsatellite loci, showing the relationships between 23 subpopulations of white spruce. CD, conifer‐dominated; MW, mixed‐wood; CON, unharvested control; 75R, 75% retention; 50R, 50% retention; 20R, 20% retention; 10R, 10% retention; CCT, clearcut.

A neighbor‐joining tree, based on Nei's (1978) genetic distances from 10 microsatellites, separated the 23 subpopulations in five groups of 2, 4, 3, 4, and 7 subpopulations, with three subpopulations grouping independently (Fig. 2). This grouping was, however, unrelated to harvest treatments, but a few CD subpopulations clustered together. The low bootstrap values do not support differentiation of the subgroups. Overall, the bootstrap values ranged from 2 to 100, with an average of 31. The neighbor‐joining tree based on the chord genetic distances (Figure S1) also did not group subpopulations according to their harvest treatments and, in general, supported the population grouping obtained from the Nei's genetic distances. As Nei's genetic distances are commonly used and reported for such studies, we are reporting the neighbor‐joining tree based on Nei's (1978) genetic distances in the main manuscript (Fig. 2) and the neighbor‐joining tree based on the chord distances in the Supporting information (Figure S1).

The patterns of *F*‐statistic estimates calculated from six EST and four genomic microsatellites were consistent between themselves (Tables S8 and S13) as well as with that observed from combined 10 microsatellites (Table 5). However, *F*_IS_ and *F*_IT_ estimates calculated from the EST microsatellites were lower than those calculated from the genomic microsatellites (Tables S8 and S13). The patterns of genetic distances among subpopulations within and among harvesting treatments were consistent for the EST and genomic microsatellites (Tables S9 and S14). However, the genetic distances calculated from the genomic microsatellites were higher than those calculated from the EST microsatellites. This may be due to higher allelic diversity and wider allele frequency distribution for the genomic than for the EST microsatellites. The neighbor‐joining trees constructed from Nei's (1978) as well as chord (Cavalli‐Sforza and Edwards 1967) genetic distances separately for the six EST and four genomic microsatellite loci showed similar results where clustering of subpopulations was not related to harvest treatments (Figures S3–S6) and some subpopulations from the same forest type CD or MW tend to cluster together. The patterns were similar to those observed from 10 microsatellite loci (Fig. 2).

The isolation‐by‐distance analysis showed that the correlation between interpopulation genetic differentiation [*F*_ST_/(1−*F*_ST_)] and geographical distances (km) was *r *=* *0.002, *P *=* *0.0.301 (Figure S2).

The simulated data sets results showed that 10 microsatellite loci were as efficient as 50 or 100 in estimating genetic diversity levels and fixation indices (Table S15 and Figure S7).

### Approach 2. Genetic diversity and population structure of preharvest old‐growth and postharvest natural regeneration in the same blocks of CD and MW stands

The genetic diversity and *F* levels were similar between the preharvest old‐growth and postharvest naturally regenerated seedlings in the same treatment blocks undergone five harvesting treatments in each of CD and MW forests (Table 7). This was the case with individual subpopulations and means over harvesting treatments. None of the genetic diversity measures and *F* was significantly different (*P *>* *0.05) between the preharvest and postharvest subpopulations and between the CD and MW forest types (Table S16).

**Table 7:** Genetic diversity parameters for preharvest pristine old‐growth and postharvest natural regeneration of white spruce in the same treatment blocks based on four genomic microsatellite loci. Values in parentheses are standard errors

Forest type	Harvesting treatment	Subpopulation	*A*_T_		*A*		*A*_e_		*A*_R_		*H*_o_		*H*_e_		*F*	
			Pre	Post	Pre	Post	Pre	Post	Pre	Post	Pre	Post	Pre	Post	Pre	Post
*(1) Preharvest old‐growth and postharvest natural regeneration (individual subpopulation)*																
CD	75R	E921	71	73	17.75	18.25	10.27	11.41	17.43	17.94	0.729	0.705	0.895	0.909	0.185	0.224
CD	50R	E920	75	77	18.75	19.25	12.66	11.43	18.50	18.91	0.693	0.814	0.916	0.904	0.243	0.101
CD	20R	E919	70	70	17.50	17.50	11.20	11.40	17.23	17.24	0.700	0.707	0.904	0.907	0.224	0.218
CD	10R	E917	70	70	17.50	17.50	11.06	11.66	17.37	17.25	0.763	0.743	0.904	0.904	0.154	0.175
CD	CCT	E922	70	64	17.50	16.00	10.95	10.65	17.19	15.83	0.771	0.643	0.902	0.898	0.146	0.281
MW	75R	E912	71	71	17.75	17.75	11.35	12.12	17.44	17.49	0.743	0.679	0.907	0.909	0.180	0.251
MW	50R	E911	73	72	18.25	18.00	12.45	12.06	18.02	17.76	0.719	0.636	0.915	0.909	0.212	0.299
MW	20R	E910	70	76	17.50	19.00	11.17	12.20	17.29	18.71	0.704	0.734	0.909	0.916	0.225	0.198
MW	10R	E913	66	73	16.50	18.25	11.36	11.97	16.27	17.97	0.707	0.586	0.910	0.909	0.223	0.356
MW	CCT	E914	71	75	17.75	18.75	11.62	11.92	17.55	18.52	0.740	0.712	0.913	0.910	0.188	0.215
*(2) Means for the preharvest old‐growth and postharvest natural regeneration (harvesting intensity wise)*																
	75R		71.0	72.0	17.80	18.00	10.81	11.77	17.44	17.72	0.736	0.692	0.901	0.909	0.183	0.238
	50R		74.0	74.5	18.50	18.63	12.56	11.75	18.26	18.34	0.706	0.725	0.916	0.907	0.228	0.200
	20R		70.0	73.0	17.50	18.25	11.19	11.80	17.26	17.98	0.702	0.721	0.907	0.912	0.225	0.208
	10R		68.0	71.5	17.00	17.88	11.21	11.82	16.82	17.61	0.735	0.665	0.907	0.907	0.189	0.266
	CCT		70.5	69.5	17.60	17.38	11.29	11.29	17.37	17.18	0.756	0.678	0.908	0.904	0.167	0.248
*(3) Overall means for the preharvest old‐growth and postharvest natural regeneration*																
			71.0	72.0	17.68	18.03	11.41	11.68	17.43	17.76	0.727	0.696	0.907	0.907	0.198	0.232
					(0.563)	(0.779)	(0.434)	(0.554)			(0.019)	(0.018)	(0.004)	(0.004)	(0.022)	(0.021)

Details of the harvesting treatments are provided in Table 1. *N*, number of samples; *A*_T_, total number of alleles; *A*, mean number of alleles per locus; *A*_e_, effective number of alleles per locus; *A*_R_, allelic richness; *H*_o_, mean observed heterozygosity; *H*_e_, mean expected heterozygosity; anova showed insignificant differences between preharvest pristine old‐growth and postharvest natural regeneration for all seven parameters (Table S16).

The mean *F*_IS_, *F*_IT,_ and *F*_ST_ estimates were slightly higher for the postharvest seedling subpopulations than for the preharvest old‐growth subpopulations (Table 8); however, the differences were not statistically significant (*P *>* *0.05). The mean *F*_IS_ was 0.198 for the preharvest old‐growth populations and 0.232 for the postharvest seedling subpopulations, with an overall mean of 0.215 (Table 8). These *F*_IS_ values were significantly different from 0 (*P *>* *0). The mean *F*_ST_ was 0.018 for the preharvest old‐growth populations and 0.023 for the postharvest seedling subpopulations, with an overall mean of 0.028 (Table 8). The indirect gene flow rates were high in the preharvest and postharvest subpopulations (Table 8).

**Table 8:** Mean *F*‐statistic estimates (SE) and indirect gene flow rates (NM) calculated for all subpopulations combined and separately for pre‐ and postharvest subpopulations based on four genomic microsatellite loci

Forest type	No. of subpopulations	*F*_IS_	*F*_IT_	*F*_ST_	*N*_M_
All subpopulations	20	0.215 (0.051)	0.236 (0.052)	0.028 (0.004)	8.68
Preharvest	10	0.198 (0.064)	0.212 (0.064)	0.018 (0.001)	13.64
Postharvest	10	0.232 (0.044)	0.249 (0.044)	0.023 (0.002)	10.62

*N*_M_: indirect migration rates calculated from *F*_ST_ estimates as *F*_ST_ = 1/4N_M_ + 1 (Crow and Aoki 1984).

## Discussion

### Overall genetic diversity and population structure

Our study suggests that white spruce at the EMEND project site in northern Alberta has high levels of microsatellite genetic diversity. Overall, genetic diversity at the EST microsatellite loci could be considered as moderate, whereas genetic diversity at the genomic microsatellite loci as very high. On average, allelic diversity at the genomic microsatellites was three times that at the EST microsatellites, suggesting that genomic microsatellites may have almost three times higher mutation rate than that of EST microsatellites. Also, lower allelic diversity at the EST microsatellites may be due to selection constraints on functional genes they are part of. It is well known that sequences of functional genes tend to be conserved. Our results from 23 subpopulations confirm the previously reported results for 10 of these subpopulations (Rajora et al. 2005) that white spruce in CD and MW forest types has similar levels of genetic diversity.

Consistent deficiency of heterozygotes relative to the Hardy–Weinberg expectation was observed in all of the studied subpopulations, and *F*_IS_ values were significantly different from 0. The *F*_IS_ values observed in this study are very similar to those observed for white spruce populations from Saskatchewan based on the same microsatellite markers (mean *F*_IS_ = 0.209). Higher than expected homozygosity may be due to inbreeding and selection against heterozygotes. White spruce has significant levels of self‐fertilization (6.2%) and biparental inbreeding (3.2%) (O'Connell et al. 2006). Selection against heterozygotes at five allozyme loci was evident in white spruce from Alberta (Rajora and Dancik 2000). The heterozygote deficiency may also result from nondetection of heterozygotes for null alleles, resulting in artificial inflation of homozygotes. However, we did not find any evidence for the presence of null alleles in the studied microsatellite loci. Heterozygote deficiency appears to be a common phenomenon in white spruce as similar results were reported for white spruce populations from Alberta (Rajora and Dancik 2000), Quebec (Tremblay and Simon 1989), and Alaska (Alden and Loopstra 1987) based on allozyme studies. However, Namroud et al. (2008), using SNP markers, reported significant excess of heterozygotes in three populations and significant excess of homozygotes in one population of white spruce sampled from Quebec. Because *F*_IS_ values were heterogeneous among the 10 SSR loci (Table S4), the effects of inbreeding causing an excess of homozygotes could be discounted because inbreeding would affect all loci. We do not exactly know the cause of deficiency of heterozygotes in the studied populations. Further investigations are required to address this issue. Overall *F*_ST_ of 0.032 suggests that most (~97%) of the genetic variation resides among individuals within subpopulations and only about 3% among populations.

### Genetic impacts of harvesting of increased intensities

The results of our study from both approaches demonstrate that genetic diversity and inbreeding levels of white spruce are similar between the preharvest old‐growth and postharvest naturally regenerated seedling populations after harvesting of five intensities. Therefore, contrary to our expectation, our study reveals that harvesting of increasing intensities of 25% cut to clearcut in 10‐ha blocks has no negative impacts on genetic diversity and fixation index of white spruce. As such, evolutionary potential for white spruce was maintained in the postharvest naturally regenerated populations.

#### Interplay of evolutionary forces: bottleneck, genetic drift, mating system, and gene flow

Harvesting creates bottlenecks in population size, which can reduce genetic diversity in postbottleneck populations by affecting genetic drift, gene flow, and mating system (Nei et al. 1975). Bottlenecks can enhance genetic drift and inbreeding and curtail gene flow in the postbottleneck populations. The genetic drift depletes allelic richness and heterozygosity, whereas inbreeding reduces heterozygosity. Allelic diversity reduces faster than heterozygosity due to population bottleneck (Nei et al. 1975; Buchert et al. 1997; Rajora et al. 2000). However, we did not find significant negative impacts of increasing harvesting intensities on allelic richness and heterozygosity measures. Clearcut creates the highest population‐size bottleneck among all five harvesting intensities studied. Thus, the highest negative genetic impacts were expected in post‐clearcut natural regeneration. However, contrary to our expectation, genetic diversity and *F* in the post‐clearcut regenerated populations were similar to those of the preharvest old‐growth populations.

Inbreeding coefficients (*F*_IS_), genetic differentiation (*F*_ST_), and genetic distances were similar among preharvest old‐growth and postharvest naturally regenerated seedling subpopulations. In addition, grouping of the subpopulations in our study from genetic distances was almost random and unrelated to forest harvesting treatments, and there was low bootstrap support for any of the grouping. These results suggest that harvesting of five intensities had not altered the inbreeding levels, genetic constitution, and population structure of the postharvest populations.

Overall, no negative impacts of harvesting of increasing intensity resulting from genetic drift, inbreeding, and fragmentation were observed. Apparently, the potential impacts of harvesting bottlenecks on genetic drift reducing allelic diversity and changing allele frequencies due to the reduction in population size, increased inbreeding due to reduction in stand density and fragmentation were counterbalanced by predominant outcrossing mating system of white spruce and high gene flow to the postharvest stands. The indirect estimates of the number of migrants (*N*_M_) calculated from the *F*_ST_ estimates in the studied white spruce populations were high (7.6–13.6; Tables 5 and 8). The *N*_M_ of 1 is sufficient to counterbalance the negative effect of genetic drift. White spruce also has high inbreeding depression, where selection against inbreeds occurs at a very early stage starting from embryo development (see O'Connell et al. 2006, 2007). All of these factors would assist in maintaining genetic diversity in the postharvest seedling populations.

Long‐distance pollen and seed dispersal can occur in white spruce. In a landscape fragmented by agriculture, 87.1% of white spruce seeds were sired by pollen from at least 250 to 3000 m away (O'Connell et al. 2007). White spruce regenerates from seed (Nienstaedt and Zasada 1990), and seed may travel from a few meters to 250 meters or more (Youngblood and Max 1992; Stewart et al. 1998). At our study sites, the distance between residual trees and between harvested blocks and unharvested surrounding forest was less than or within this range. Therefore, high pollen and seed dispersal between and within the subpopulations and from the surrounding white spruce stands may have contributed significantly to maintaining high genetic diversity and homogeneity in postharvest naturally regenerated seedling populations and low differentiation between them and with the preharvest gene pool. White spruce is continuously distributed in the CD and MW forests at and around the study site. The harvested blocks are surrounded by unharvested CD and MW forests with the same physical structure as the harvested stands. The postharvest residual and surrounding white spruce cover likely have genetic constitution similar to the preharvest gene pool.

The Mantel test also suggested that the sampled white spruce populations are well connected and do not show significant IBD. Local adaptation and genetic drift are two important forces that can cause IBD (Slatkin 1993; Mimura and Aitken 2007). IBD usually depends on equilibrium between gene flow or migration and genetic drift (Crow and Aoki 1984; Slatkin 1993). Generally, populations will only begin to show IBD when gene flow is restricted and a sufficient amount of time has passed so that random genetic drift alters allele frequencies. Populations need to be sufficiently small for this to occur. However, this was not the case with the populations sampled in this study.

Overall, our study demonstrates that harvesting of five intensities had no negative effect on genetic diversity, inbreeding levels, differentiation, and population genetic structure of white spruce in the CD and MW forests. This may indicate that the evolutionary processes, gene flow, mating system, genetic drift, and selection were maintained at the preharvest levels.

#### Clearcut harvesting

Genetic diversity in the post‐clearcut regenerated populations was similar to that of the preharvest old‐growth populations. Within the EMEND project design, clearcut blocks retained 2% of the original trees; therefore, it is possible that the residual trees may have received pollen from surrounding white spruce trees. Also, white spruce seed dispersal from adjacent partially harvested blocks and surrounding white spruce forest would have been high to the clearcut blocks. During sample collection, we noticed that white spruce seedlings in the clearcut, 10% retention, or 20% retention blocks were more abundant and healthy in comparison with 75% retention or unharvested blocks. The blocks with lower tree retention were more exposed to sunlight, and soils of these blocks also had more disturbances due to harvesting operations; thus, these blocks may have provided a better environment for seed germination, and growth and survival of seedlings. All of these factors result in high density of white spruce seedlings in clearcut blocks, which would assist in maintaining genetic diversity. Solarik et al. (2012a,b) reported that in white spruce, natural regeneration can be increased substantially by clearcutting or partial harvesting and passive site preparation provided that sufficient number of seed trees is maintained. Lieffers et al. (2009) suggested that in the CD stands, 25–30% retention of mature white spruce trees, and in the deciduous dominated stands, 50% tree retention could improve white spruce seedling establishment. Therefore, white spruce could be managed under clearcut and natural regeneration system provided the cut blocks are not large and are surrounded by local white spruce cover.

#### Ecological conditions and genetic diversity

Ecosite conditions can affect genetic diversity of the inhabitant species by affecting its demography and selection pressures. The EMEND project site has quite uniform ecosite and environmental conditions over different forest types, replicates, and harvesting treatments (http://www.emendproject.org). The soils of the EMEND experimental site are usually well‐drained luvisolic, and soil properties (e.g., soil parent material, soil formation, and soil classification) over the entire EMEND experimental site are equally consistent (Kishchuk 2004). Nitrogen contents and availability were found to be consistent in 50% and 20% retention and clearcuts of CD and MW stands 4 years after harvesting (Jerabkova et al. 2006). Microbial community was fairly consistent among variable retention harvesting (Hannam et al. 2006). Therefore, soil properties and other ecosite conditions, which are quite uniform over the EMEND project site, may not have any effect on the genetic diversity of the subpopulations sampled from different treatment blocks.

### Conifer‐dominated versus mixed‐wood forests

Higher negative genetic effects were expected for white spruce with harvesting of increasing intensities in MW than in CD stands because of higher number of white spruce trees per unit area in CD stands as compared to MW stands, and higher barriers to white spruce gene flow in MW than in CD stands (Rajora et al. 2005). However, contrary to our expectation, our study demonstrated that white spruce in the CD and MW forests responded similarly to harvesting treatments by maintaining genetic diversity in the postharvest seedlings from their parental old‐growth populations. This observation may be due to the fact that CD and MW stands in this study exist adjacent to each other and a high gene flow is expected between the stands. However, clustering of some postharvest CD subpopulations together (Fig. 2) may suggest that these populations may have had somewhat similar genetic constitution. Saetre et al. (1997) reported that in comparison with CD stands, MW stands provide more heterogeneous conditions with better light transmittance and litter quality. This may influence the germination and survival of young seedlings of white spruce and other understory species. We assumed that blocks under CD stands had an advantage of higher seed rain, but the blocks under MW stands had an advantage of better seed germination conditions. This may be another contributing factor to our observation of maintenance of similar genetic diversity in pre‐ and postharvest of CD and MW white spruce stands.

Our results showing no negative genetic impacts of harvesting of increased intensities are similar to the results reported for Douglas‐fir (Adams et al. 1998) and amabilis fir (El‐Kassaby et al. 2003), where shelterwood cut, patch‐cut/group selection cut, and clearcut harvesting showed no significant negative genetic impacts. However, these studies did not cover all harvesting intensities, forest types, replicates, and postharvest natural regeneration as our study did. On the other hand, our results are in contrast from those reported for eastern white pine, where shelterwood cut (25–30% tree removal) had no significant negative impact on genetic diversity (Marquardt et al. 2007; Rajora et al. unpublished data), but removal of 75% of eastern white pine trees had significant negative impact on genetic diversity (Buchert et al. 1997; Rajora et al. 2000). The differences between white spruce and eastern white pine may be due to differences in their distribution, demography, study‐site location, reproductive and biological characteristics. Both white spruce and eastern white pine are widely distributed species with high genetic diversity, predominantly outbreeding mating system and inbreeding depression. White spruce in our present study was sampled from its central continuous range, whereas eastern white pine stands in Buchert et al. (1997) and Rajora et al. (2000) were marginal, small, and somewhat isolated. Also, eastern white pine does not maintain viable seed bank in soil.

Heterozygosity is expected to increase with age in conifers (e.g., Ledig et al. 1983; Sharma et al. 2007) if selection against inbreds continues over time. The age of the postharvest regeneration sampled in our study was around 4 years. It is quite possible that heterozygosity of the postharvest naturally regenerated subpopulations may increase with age. Thus, it would be wise to assess genetic diversity of the postharvest subpopulations periodically.

### Marker number and marker type

Our simulated microsatellite data analysis suggests that 10 microsatellite markers are as efficient to detect the level of genetic diversity and fixation index as 50 or 100 microsatellite markers are (Table S15 and Figure S7). We expect that the SNP markers from neutral sequences as well as from adaptive genes involved in height, adaptation, and other traits should provide the same patterns because white spruce trees were harvested randomly rather than based on their age, height, or other adaptive traits. No differential selective pressures among different harvesting treatments are evident that would result in divergent selection. We also think that being hypervariable markers, microsatellites should be able to pick subtle genetic differences among harvesting treatments more efficiently than biallelic SNP markers.

### Practical implications

Among different commercial forest harvesting methods, clearcutting is the most prevalent in the region. However, other EMEND studies suggest that variable retention harvest better preserves the boreal forest ecosystems and is an increasingly popular management alternative to clearcutting (Lieffers et al. 2009; Solarik et al. 2010,2012a,b). The variable retention systems used in the EMEND experiment are close to commercial harvesting practices: 75R = first shelterwood; 50R = second shelterwood; 10R/20R = seed tree cut; and clearcut. Our study suggests that white spruce could be managed without adverse effect on genetic diversity and population structure under any of these partial and clearcut harvesting systems provided there is surrounding natural white spruce cover. However, the operational clearcut blocks are several times larger than the 10‐ha blocks that we studied. We would suggest leaving some buffer strips of local forest cover between clearcuts blocks of 10 ha so that genetic diversity could be maintained. Other EMEND studies have suggested that variable retention systems better preserve boreal ecosystem (Lieffers et al. 2009; Solarik et al. 2010,2012a,b. Our study supports using such systems as our study demonstrates that the variable retention systems used at the EMEND project site do not adversely affect genetic diversity.

## Conclusions

Harvesting of increasing intensities from 25 to 100% harvesting of forest did not have negative genetic impacts in white spruce. Evolutionary processes in the postharvest populations and their evolutionary potential were apparently maintained at the preharvest levels. White spruce in CD and MW forests responds similarly to harvesting of increasing intensities with no negative impacts on its genetic diversity and population structure. The potential negative genetic impacts of genetic drift and inbreeding resulting from harvesting bottlenecks were apparently counterbalanced by high gene flow and predominantly outcrossing in the studied populations. White spruce could be managed under partial (variable retention) and clearcut harvesting and natural regeneration system in 10‐ha blocks surrounded by natural white spruce forest without any genetic degradation. However, by taking into account the results of other studies at the EMEND site, we suggest variable retention harvesting systems over clearcutting for ecologically sustainable forest management. This is the first study of its kind examining the genetic impacts of harvesting of five increasing intensities in a forest tree by using a long‐term well‐designed controlled, replicated experimental harvesting experiment and comparing genetic impacts of harvesting in a dominant boreal conifer species in CD and MW forests. Also, it is the first study where two approaches (adjacent control and harvesting treatments; and pre‐ and postharvest population genetic characteristics) have been used at the same time at the same sites, providing more scientifically sound outcomes. Our study results have significant implications and applications for the conservation and sustainable management of white spruce genetic resources and should be widely applicable to other tree species. Our study addresses the broad central forest management question how forest harvesting and regeneration practices can best maintain biodiversity, forest structure and function, and ecosystem integrity.

In this study, four‐year‐old or younger naturally regenerated populations of white spruce were sampled. We suggest resampling the populations from the same treatment blocks periodically and genotyping using a larger number of both neutral and adaptive markers. This information would be helpful in better understanding the long‐term impacts of harvesting treatments on evolutionary processes of white spruce.

## Authors' contributions

MSF was involved in field sampling, partial microsatellite genotyping, data analysis under the directions of OPR, initial manuscript draft preparation and revision. OPR developed the study concept and experimental design, provided overall study direction and supervision, and contributed to data interpretation, and manuscript writing and revision.

## Data archiving statement

We have submitted the data underlying the reported results to Dryad data repository. doi:10.5061/dryad.hj06k.

## Supporting information


**Table S1.** Genetic diversity parameters and fixation index (*F*) of unharvested control and post‐harvest natural regeneration subpopulations of white spruce in the conifer‐dominated forest based on 10 microsatellite loci.
**Table S2.** Genetic diversity parameters and fixation index (*F*) of unharvested control and post‐harvest natural regeneration subpopulations of white spruce in the mixed‐wood forest based on 10 microsatellite loci.
**Table S3.** Analysis of variance results for different genetic diversity parameters for testing the differences due to harvesting treatments and forest types (Approach 1).
**Table S4.** Locus‐wise mean *F*‐statistic estimates calculated for 23 subpopulations.
**Table S5.** Genetic diversity parameters and fixation index (*F*) of unharvested control and post‐harvest natural regeneration Subpopulations of white spruce in the conifer‐dominated forest based on six EST microsatellite loci.
**Table S6.** Genetic diversity parameters and fixation index (*F*) of unharvested control and post‐harvest natural regeneration Subpopulations of white spruce in the mixed‐wood forest based on six EST microsatellite loci.
**Table S7.** Overall means for genetic diversity parameters and fixation index for unharvested control and post‐harvest natural regeneration of white spruce in the conifer‐dominated and mixed‐wood forests based on six EST microsatellite loci.
**Table S8.** Mean *F*‐statistic estimates from six EST microsatellite loci, calculated over all subpopulations and separately by harvesting treatments.
**Table S9.** Mean and (range) of genetic distances (Nei 1978) among (below the diagonal) and within (on the diagonal) harvesting treatments based on six EST microsatellite loci.
**Table S10.** Genetic diversity parameters and fixation index for unharvested control and post‐harvest natural regeneration of white spruce in the conifer‐dominated forest based on four genomic microsatellite loci.
**Table S11.** Genetic diversity parameters and fixation index for unharvested control and post‐harvest natural regeneration of white spruce in the mixed‐wood forest based on four genomic microsatellite loci.
**Table S12.** Overall means of genetic diversity parameters and fixation index for unharvested control and post‐harvest natural regeneration of white spruce in the conifer‐dominated and mixed‐wood forests based on four genomic microsatellite loci.
**Table S13.** Mean *F‐*statistic estimates from four genomic microsatellite loci, calculated over all subpopulations and separately by harvesting treatments.
**Table S14.** Mean and (range) of genetic distances (Nei 1978) among (below the diagonal) and within (on the diagonal) harvesting treatments based on four genomic microsatellite loci.
**Table S15.** Estimation of genetic diversity measures and inbreeding coefficient calculated for 10, 50, and 100 microsatellite markers from the simulated datasets.
**Table S16.** Analysis of variance results for different genetic diversity parameters for testing the differences due to harvesting treatments and forest types (pre‐ versus post‐harvest) (Approach 2).
**Figure S1.** A neighbor‐joining tree, based on Cavalli‐Sforza and Edwards' chord distance *D*_C_ (Cavalli‐Sforza and Edwards 1967) from 10 microsatellite loci, showing the relationships among 23 subpopulations of white spruce.
**Figure S2.** Relationship between genetic differentiation (*F*_*ST*_) and geographical distance.
**Figure S3.** A neighbor‐joining tree, based on genetic distances (Nei 1978) from six EST‐based microsatellite loci, showing the relationships among 23 subpopulations of white spruce.
**Figure S4.** A neighbor‐joining tree, based on Cavalli‐Sforza and Edwards' chord distance *D*_C_ (Cavalli‐Sforza and Edwards 1967) for six EST microsatellite loci, showing the relationships among 23 subpopulations of white spruce.
**Figure S5.** A neighbor joining tree, based on genetic distances (Nei 1978) from four genomic microsatellite loci, showing the relationships among 23 subpopulations of white spruce.
**Figure S6.** A neighbor‐joining tree, based on Cavalli‐Sforza and Edwards' chord distance *D*_C_ (Cavalli‐Sforza and Edwards 1967) for four genomic microsatellite loci, showing the relationships among 23 subpopulations of white spruce.

## References

[eva12064-bib-0001] Adams, W. T. , J. Zuo , J. Y. Shimizu , and J. C. Tappeiner . 1998 Impact of alternative regeneration methods on genetic diversity in coastal Douglas‐fir. Forest Science 44:390–396.

[eva12064-bib-0002] Alden, J. , and C. Loopstra . 1987 Genetic diversity and population structure of *Picea glauca* on an altitudinal gradient in interior Alaska. Canadian Journal of Forest Research 17:1519–1526.

[eva12064-bib-0003] Balloux, F. 2001 Easypop (Version 1.7): a computer program for population genetics simulations. Journal of Heredity 92:301–302. 11447253 10.1093/jhered/92.3.301

[eva12064-bib-0004] Bashalkhanov, S. , and O. P. Rajora . 2008 Protocol: a high‐throughput DNA extraction system suitable for conifers. Plant Methods 4:20. 18673554 10.1186/1746-4811-4-20 PMC2518145

[eva12064-bib-0005] Beaulieu, J. , M. Deslauriers , and Y. Bergeron . 2003 Are old‐growth forests from the Abitibi region an important reservoir of genetic diversity for white spruce in Quebec? Proceedings of the Symposium of the North American Forest Commission, Forest Genetic Resources and Silviculture Working Groups, and the International Union of Forest Research Organizations (IUFRO), Quebec City, Canada, September 21, 2003, pp. 51–66.

[eva12064-bib-0006] Booy, G. , R. J. J. Hendriks , M. J. M. Smulders , J. M. van Groenendae , and B. Vosman . 2000 Genetic diversity and the survival of populations. Plant Biology 2:379–395.

[eva12064-bib-0007] Buchert, G. P. , O. P. Rajora , J. V. Hood , and B. P. Dancik . 1997 Effects of harvesting on genetic diversity in old‐growth eastern white pine in Ontario, Canada. Conservation Biology 11:747–758.

[eva12064-bib-0008] Buiteveld, J. , G. G. Vendramin , S. Leonardi , K. Kamer , and T. Geburek . 2007 Genetic diversity and differentiation in European beech (*Fagus sylvatica* L.) stands varying in management history. Forest Ecology and Management 247:98–106.

[eva12064-bib-0009] Carneiro, F. S. , A. E. B. Lacerda , M. R. Leme , R. Gribel , M. Kanashiro , L. H. O. Wadt , and A. M. Sebbenn . 2011 Effects of selective logging on the mating system and pollen dispersal of *Hymenaea courbaril* L. (leguminosae) in the Eastern Amazon as revealed by microsatellite analysis. Forest Ecology and Management 262:1758–1765.

[eva12064-bib-0010] Cavalli‐Sforza, L. L. , and A. W. F. Edwards . 1967 Phylogenetic analysis: models and estimation procedures. American Journal of Human Genetics 19:233–257. 6026583 PMC1706274

[eva12064-bib-0011] Cloutier, D. , M. Kanashiro , A. Y. Ciampi , and D. J. Schoen . 2007 Impacts of selective logging on inbreeding and gene dispersal in a Amazonian tree population of *Carapa guianensis* Aubl. Molecular Ecology 16:797–809. 17284212 10.1111/j.1365-294X.2006.03193.x

[eva12064-bib-0012] Crow, J. F. , and K. Aoki . 1984 Group selection for a polygenic behavioural trait: estimating the degree of population subdivision. Proceedings of the National Academy of Sciences of the United States of America 81:6073–6077. 6592602 10.1073/pnas.81.19.6073 PMC391861

[eva12064-bib-0013] El‐Kassaby, Y. A. , B. G. Dunsworth , and J. Krakowski . 2003 Genetic evaluation of alternative silvicultural systems in coastal montane forests: western hemlock and amabilis fir. Theoretical and Applied Genetics 107:598–610. 12750773 10.1007/s00122-003-1291-3

[eva12064-bib-0014] Felsenstein, J. 2004 Inferring Phylogenies. Sinauer Associates, Sunderland, MA.

[eva12064-bib-0015] Finkeldey, R. , and M. Ziehe . 2004 Genetic implications of silvicultural regimes. Forest Ecology and Management 197:231–244.

[eva12064-bib-0016] Gaudet, J. 1995 FSTAT version 1.2: a computer program to calculate F‐statistics. Journal of Heredity 86:485–486.

[eva12064-bib-0017] Glaubitz, J. C. , J. C. Murrell , and G. F. Moran . 2003a Effects of native forest regeneration practices on genetic diversity in *Eucalyptus consideniana* . Theoretical and Applied Genetics 107:422–431. 12748762 10.1007/s00122-003-1262-8

[eva12064-bib-0018] Glaubitz, J. C. , H. X. Wu , and G. F. Moran . 2003b Impacts of silviculture on genetic diversity in the native forest species *Eucalyptus sieberi* . Conservation Genetics 4:275–287.

[eva12064-bib-0019] Gömöry, D. 1992 Effect of stand origin on the genetic diversity of Norway spruce (*Picea abies* Karst.) populations. Forest Ecology and Management 54:215–223.

[eva12064-bib-0020] Hannam, K. , S. A. Quideau , and B. E. Kishchuk . 2006 Forest floor microbial communities in relation to stand composition and timber harvesting in northern Alberta. Soil Biology and Biochemistry 38:2565–2575.

[eva12064-bib-0021] Hodgetts, R. B. , M. A. Aleksiuk , A. Brown , C. Clark , E. Macdonald , S. Nadeem , and P. D. Khasa . 2001 Development of microsatellite markers for white spruce (*Picea glauca*) and related species. Theoretical and Applied Genetics 102:1252–1258.

[eva12064-bib-0022] Hosie, R. C. 1979 Native Trees of Canada. Fitzhenry and Whiteside Ltd, Don Mills, ON.

[eva12064-bib-0023] Jerabkova, L. , C. E. Prescott , and B. E. Kishchuk . 2006 Effect of variable‐retention harvesting on soil nitrogen availability in boreal mixedwood forests. Canadian Journal of Forest Research 36:3029–3038.

[eva12064-bib-0024] Kishchuk, B. E. 2004 Soils of the Ecosystem Management Emulating Natural Disturbance (EMEND) experimental area, northwestern Alberta. Nat. Resour. Can., Can. For. Serv., North. For. Cent., Edmonton, Alberta. Inf. Rep. NOR‐X‐397.

[eva12064-bib-0025] Krakowski, J. , and Y. A. El‐Kassaby . 2003 Impacts of alternative silviculture systems on mating systems and genetic diversity of Forest tree species. Proceedings of the Symposium of the North American Forest Commission, Forest Genetic Resources and Silviculture Working Groups, and the International Union of Forest Research Organizations (IUFRO), Quebec City, Canada, September 21, 2003, pp. 75–87.

[eva12064-bib-0026] Ledig, F. T. , R. P. Guries , and B. A. Bonefeld . 1983 The relation of growth to heterozygosity in pitch pine. Evolution 37:1227–1238. 28556005 10.1111/j.1558-5646.1983.tb00237.x

[eva12064-bib-0027] Lieffers, V. , D. Sidders , T. Gradowski , S. Landhäusser , B. Frey , A. Munson , T. Keddy et al. 2009 Spruce and aspen regeneration following variable retention harvests at EMEND. Sustainable Forest Management Network Research Note Series No. 41.

[eva12064-bib-0029] Marquardt, P. E. , C. S. Echt , B. K. Epperson , and D. M. Pubanz . 2007 Genetic structure, diversity, and inbreeding of eastern white pine under different management conditions. Canadian Journal of Forest Research 37:2652–2662.

[eva12064-bib-0030] Mimura, M. , and S. N. Aitken . 2007 Adaptive gradients and isolation‐by‐distance with postglacial migration in *Picea sitchensis* . Heredity 99:22–24. 10.1038/sj.hdy.6800987 17487214

[eva12064-bib-0031] Namroud, M. C. , J. Beaulieu , N. Juge , J. Laroche , and J. Bousquet . 2008 Scanning the genome for gene single nucleotide polymorphisms involved in adaptive population differentiation in white spruce. Molecular Ecology 17:3599–3613. 18662225 10.1111/j.1365-294X.2008.03840.x PMC2613251

[eva12064-bib-0032] Neale, D. B. 1985 Genetic implications of shelterwood regeneration of Douglas‐fir in southwestern Oregon. Forest Science 31:995–1005.

[eva12064-bib-0033] Neale, D. B. , and W. T. Adams . 1985 The mating system in natural and shelter wood stands in Douglas‐fir. Theoretical and Applied Genetics 71:201–207. 24247383 10.1007/BF00252056

[eva12064-bib-0034] Nei, M. 1978 Estimation of average heterozygosity and genetic distance from a small number of individuals. Genetics 89:583–590. 17248844 10.1093/genetics/89.3.583 PMC1213855

[eva12064-bib-0035] Nei, M. , T. Maruyama , and R. Chakraborty . 1975 The bottleneck effect and genetic variability in populations. Evolution 29:1–10. 28563291 10.1111/j.1558-5646.1975.tb00807.x

[eva12064-bib-0036] Ng, K. K. S. , S. L. Lee , and S. Ueno . 2009 Impact of selective logging on genetic diversity of two tropical tree species with contrasting breeding systems using direct comparison and simulation methods. Forest Ecology and Management 257:107–116.

[eva12064-bib-0037] Nienstaedt, H. , and J. C. Zasada . 1990 *Picea glauca* (Moench) Voss. White spruce In Burns R. M., and Honkala B. H., eds. Silvics of North America. Vol. 1. Conifers, pp. 204–226. U.S. Department of Agriculture Handb. 654. United States Department of Agriculture, Washington, DC, USA.

[eva12064-bib-0038] O'Connell, L. M. , A. Mosseler , and O. P. Rajora . 2006 Impacts of forest fragmentation on the mating system and genetic diversity of white spruce (*Picea glauca*) at the landscape level. Heredity 97:418–426. 16912700 10.1038/sj.hdy.6800886

[eva12064-bib-0039] O'Connell, L. M. , A. Mosseler , and O. P. Rajora . 2007 Extensive long‐distance pollen dispersal in a fragmented landscape maintains genetic diversity in white spruce. Journal of Heredity 98:640–645. 17981919 10.1093/jhered/esm089

[eva12064-bib-0040] van Oosterhout, C. , W. F. Hutchinson , D. P. M. Wills , and P. Shipley . 2004 Micro‐checker: software for identifying and correcting genotyping errors in microsatellite data. Molecular Ecology Notes 4:535–538.

[eva12064-bib-0041] Peakall, R. , and P. E. Smouse . 2006 GENALEX6: genetic analysis in Excel. Population genetic software for teaching and research. Molecular Ecology Notes 6:288–295.

[eva12064-bib-0042] Perry, D. J. , and J. Bousquet . 2001 Genetic diversity and mating system of post‐fire and post‐harvest black spruce: an investigation using co dominant sequence‐tagged‐site (STS) markers. Canadian Journal of Forest Research 31:32–40.

[eva12064-bib-0043] Rajora, O. P. 1999 Genetic biodiversity impacts of silvicultural practices and phenotypic selection in white spruce. Theoretical and Applied Genetics 99:954–961.

[eva12064-bib-0044] Rajora, O. P. , and B. P. Dancik . 2000 Population genetic structure, variation, and evolution of Engelmann spruce, white spruce and their putative natural hybrid complex in Alberta. Canadian Journal of Botany 78:768–780.

[eva12064-bib-0045] Rajora, O. P. , and A. Mosseler . 2001a Challenges and opportunities for conservation of forest genetic resources. Euphytica 118:197–212.

[eva12064-bib-0046] Rajora, O.P. , and A. Mosseler . 2001b Molecular markers in conservation, restoration and sustainable management of forest genetic resources In Muller‐Starck G., and Schubert R., eds. Genetic Response of Forest Systems to Changing Environmental Conditions. Kluwer Academic Publishers, Dordrecht, The Netherlands *Forestry Sciences* 70:187–201.

[eva12064-bib-0047] Rajora, O. P. , and S. A. Pluhar . 2003 Genetic diversity impacts of forest fires, forest harvesting and alternative reforestation practices in black spruce (*Picea mariana*). Theoretical and Applied Genetics 106:1213–1224. 12748771 10.1007/s00122-002-1169-9

[eva12064-bib-0048] Rajora, O. P. , M. H. Rahman , G. P. Buchert , and B. P. Dancik . 2000 Microsatellite analysis of genetic effects of harvesting in old‐growth eastern white pine (*Pinus strobus*) in Ontario, Canada. Molecular Ecology 9:339–348. 10736031 10.1046/j.1365-294x.2000.00886.x

[eva12064-bib-0049] Rajora, O. P. , M. H. Rahman , S. Dayanandan , and A. Mosseler . 2001 Isolation, characterization, inheritance and linkage of microsatellite DNA markers in white spruce (*Picea glauca*) and their usefulness in other spruce species. Molecular and General Genetics 264:871–882. 11254135 10.1007/s004380000377

[eva12064-bib-0050] Rajora, O. P. , I. K. Mann , and Y. Z. Shi . 2005 Genetic diversity and population structure of boreal white spruce (*Picea glauca*) in pristine conifer‐dominated and mixed‐wood forest stands. Canadian Journal of Botany 83:1096–1105.

[eva12064-bib-0051] Raymond, M. , and F. Rousset . 1995 GENEPOP version 1.2: population genetics software for the exact tests and ecumenicism. Journal of Heredity 86:248–249.

[eva12064-bib-0052] Rousset, F. 1997 Genetic differentiation and estimation of gene flow from *F*‐statistics under isolation by distance. Genetics 145:1219–1228. 9093870 10.1093/genetics/145.4.1219 PMC1207888

[eva12064-bib-0053] Saetre, P. , L. S. Saetre , P. O. Brandtberg , H. Lundkvist , and J. Bengtsson . 1997 Ground vegetation competition and heterogeneity in pure Norway spruce and mixed Norway spruce‐birch stands. Canadian Journal of Forest Research 27:2034–2042.

[eva12064-bib-0054] SAS Institute Inc . 2001 SAS System for Windows, Release 8.11. SAS Institute, Inc. Cary, NC.

[eva12064-bib-0055] Sharma, K. , B. Degen , G. V. Wuehlisch , and N. B. Singh . 2007 An assessment of heterozygosity and fitness in Chir pine (*Pinus roxburghii* Sarg.) using isozymes. New Forests 34:153–162.

[eva12064-bib-0056] Slatkin, M. 1993 Isolation by distance in equilibrium and non‐equilibrium populations. Evolution 47:264–279. 28568097 10.1111/j.1558-5646.1993.tb01215.x

[eva12064-bib-0057] Solarik, K. A. , V. J. Lieffers , W. J. A. Volney , R. Pelletier , and J. R. Spence . 2010 Seed tree density, variable retention, and stand composition influence recruitment of white spruce in boreal mixedwood forests. Canadian Journal of Forest Research 40:1821–1832.

[eva12064-bib-0058] Solarik, K. A. , V. J. Lieffers , and W. J. A. Volney . 2012a The interaction between retention harvests and natural regeneration. EMEND Insights #1. p. 6.

[eva12064-bib-0059] Solarik, K. A. , W. J. Volney , V. J. Lieffers , J. R. Spence , and A. Hamann . 2012b Factors affecting white spruce and aspen survival after partial harvest. Journal of Applied Ecology. 49:145–154.

[eva12064-bib-0060] Stewart, J. D. , E. H. Hogg , P. A. Hurdle , K. J. Stadt , P. Tollestrup , and V. J. Lieffers . 1998 Dispersal of white spruce seed in mature aspen stands. Canadian Journal of Botany 76:181–188.

[eva12064-bib-0061] Thomas, B. R. , S. E. Macdonald , M. Hicks , D. L. Adams , and R. B. Hodgetts . 1999 Effects of reforestation methods on genetic diversity of lodgepole pine: an assessment using microsatellite and randomly amplified polymorphic DNA markers. Theoretical and Applied Genetics 98:793–801.

[eva12064-bib-0062] Tremblay, N. , and J. P. Simon . 1989 Genetic structure of marginal populations of white spruce as its northern limit of distribution in Nouveau‐Quebec. Canadian Journal of Forest Research 19:1371–1379.

[eva12064-bib-0064] Youngblood, A. , and T. A. Max . 1992 Dispersal of white spruce seed on Willow Island in interior Alaska. Research Paper, PNW‐RP‐443. Portland, OR: U.S. Department of Agriculture, Forest Service, Pacific Northwest Research Station p. 17.

