# Supplementary material for: Effects of harvesting of increasing intensities on genetic diversity and population structure of white spruce
Source: Evol Appl. 2013 Apr 18;6(5):778–94. doi: 10.1111/eva.12064 (PMC5779121; doi:10.1111/eva.12064)
Supplement: Supplementary file 1 — Table S1. Genetic diversity parameters and fixation index (F) of unharvested control and post‐harvest natural regeneration subpopulations of white spruce in the conifer‐dominated forest based on 10 microsatellite loci. Table S2. Genetic diversity parameters and fixation index (F) of unharvested control and post‐harvest natural regeneration subpopulations of white spruce in the mixed‐wood forest based on 10 microsatellite loci. Table S3. Analysis of variance results for different genetic diversity parameters for testing the differences due to harvesting treatments and forest types (Approach 1). Table S4. Locus‐wise mean F‐statistic estimates calculated for 23 subpopulations. Table S5. Genetic diversity parameters and fixation index (F) of unharvested control and post‐harvest natural regeneration Subpopulations of white spruce in the conifer‐dominated forest based on six EST microsatellite loci. Table S6. Genetic diversity parameters and fixation index (F) of unharvested control and post‐harvest natural regeneration Subpopulations of white spruce in the mixed‐wood forest based on six EST microsatellite loci. Table S7. Overall means for genetic diversity parameters and fixation index for unharvested control and post‐harvest natural regeneration of white spruce in the conifer‐dominated and mixed‐wood forests based on six EST microsatellite loci. Table S8. Mean F‐statistic estimates from six EST microsatellite loci, calculated over all subpopulations and separately by harvesting treatments. Table S9. Mean and (range) of genetic distances (Nei 1978) among (below the diagonal) and within (on the diagonal) harvesting treatments based on six EST microsatellite loci. Table S10. Genetic diversity parameters and fixation index for unharvested control and post‐harvest natural regeneration of white spruce in the conifer‐dominated forest based on four genomic microsatellite loci. Table S11. Genetic diversity parameters and fixation index for unharvested control and post‐harvest na [file EVA-6-778-s001.docx]

**SUPPLEMENTARY MATERIAL**

**doi: 10.1111/eva.12064**

**Effects of harvesting of increasing intensities on genetic diversity and population structure of white spruce**

Manphool S. Fageria^1, 2^, and Om P. Rajora^1*^

^1^Canadian, Genomics and Conservation Genetics Institute, Faculty of Forestry and Environmental Management, University of New Brunswick,

28 Dineen Drive, Fredericton NB E3B 5A3, Canada

^2^ Current address: Agriculture and Agri-Food Canada, 850 Lincoln Road, Fredericton, NB E3B 4Z7, Canada

## *Principal Investigator and Author for Correspondence:

## Om P. Rajora

## E-mail: [Om.Rajora@unb.ca](mailto:Om.Rajora@unb.ca)

## Telephone: (506) 458-7477

## Fax: (506) 453-3538

**Supplementary Material**

**Table S1.** Genetic diversity parameters and fixation index (*F*) of unharvested control and post-harvest natural regeneration subpopulations

of white spruce in the conifer-dominated forest based on 10 microsatellite loci.

| FT | Rep | HT | SP | A_T_ | A | A*_e_* | A_R_ | A_P_ | H*_o_* | H*_e_* | *F* |
| --- | --- | --- | --- | --- | --- | --- | --- | --- | --- | --- | --- |
| **(1) Individual subpopulation** | | | | | | | | | | | |
| CD | R1 | CON | E889 | 115 | 11.50 (2.20) | 6.46 (1.71) | 10.39 | 1 | 0.475 (0.071) | 0.637 (0.094) | 0.184 (0.064) |
| CD | R1 | 75R | E890 | 108 | 10.80 (2.35) | 7.05 (1.74) | 10.14 | 0 | 0.534 (0.083) | 0.638 (0.098) | 0.150 (0.050) |
| CD | R1 | 50R | E898 | 101 | 10.10 (2.55) | 6.94 (1.76) | 9.90 | 0 | 0.547 (0.089) | 0.681 (0.092) | 0.190 (0.038) |
| CD | R1 | 20R | E896 | 110 | 11.00 (2.03) | 6.06 (1.61) | 10.23 | 0 | 0.523 (0.071) | 0.629 (0.096) | 0.173 (0.045) |
| CD | R1 | 10R | E895 | 110 | 11.00 (2.57) | 7.68 (1.88) | 9.71 | 1 | 0.573 (0.089) | 0.679 (0.095) | 0.118 (0.071) |
| CD | R1 | CCT | E892 | 119 | 11.90 (2.15) | 6.94 (1.57) | 9.74 | 2 | 0.543 (0.072) | 0.667 (0.086) | 0.166 (0.061) |
| CD | R2 | Con | E918 | 109 | 10.90 (2.69) | 6.53 (1.78) | 10.92 | 0 | 0.509 (0.065) | 0.641 (0.100) | 0.165 (0.062) |
| CD | R2 | 75R | E921 | 121 | 12.10 (2.65) | 7.24 (1.88) | 10.61 | 1 | 0.524 (0.093) | 0.673 (0.104) | 0.232 (0.048) |
| CD | R2 | 50R | E920 | 116 | 11.60 (2.50) | 6.80 (1.93) | 9.90 | 1 | 0.596 (0.076) | 0.668 (0.083) | 0.114 (0.056) |
| CD | R2 | 20R | E919 | 107 | 10.70 (2.55) | 6.21 (1.63) | 9.45 | 1 | 0.523 (0.090) | 0.648 (0.095) | 0.149 (0.060) |
| CD | R2 | 10R | E917 | 109 | 10.90 (3.24) | 6.55 (2.00) | 10.85 | 1 | 0.540 (0.079) | 0.638 (0.091) | 0.206 (0.070) |
| CD | R2 | CCT | E922 | 106 | 10.60 (2.62) | 6.03 (1.77) | 10.36 | 1 | 0.509 (0.090) | 0.664 (0.094) | 0.227 (0.073) |
| **2) Means conifer-dominated** | | | | | | | | | | | |
|  |  | CON |  | 112.0 | 11.20 | 6.50 | 10.66 | 0.5 | 0.492 | 0.639 | 0.175 |
|  |  | 75R |  | 114.5 | 11.45 | 7.15 | 10.38 | 0.5 | 0.529 | 0.656 | 0.191 |
|  |  | 50R |  | 108.5 | 10.85 | 6.87 | 9.90 | 0.5 | 0.572 | 0.675 | 0.152 |
|  |  | 20R |  | 108.5 | 10.85 | 6.14 | 9.84 | 0.5 | 0.523 | 0.639 | 0.161 |
|  |  | 10R |  | 109.5 | 10.95 | 7.12 | 10.28 | 1.0 | 0.557 | 0.659 | 0.162 |
|  |  | CCT |  | 112.5 | 11.25 | 6.49 | 10.05 | 1.5 | 0.526 | 0.666 | 0.197 |
| **(3) Overall mean CD** | | | | 110.9 | 11.09 | 6.71 | 10.18 | 0.7 | 0.533 | 0.655 | 0.173 |

Details of the harvesting treatments (HT) are provided in Table 1. N, number of samples; A_T_, total number of alleles; A, mean number of alleles

per locus; A_e_, effective number of alleles per locus; A_R_, allelic richness; A_P_, private alleles; H_o_, mean observed heterozygosity; H_e_, mean expected heterozygosity. FT, forest type; Rep, replication; HT, harvesting treatment; SP, sub-population. Values in parentheses are standard errors.

**Table S2.** Genetic diversity parameters and fixation index (*F*) of unharvested control and post-harvest natural regeneration subpopulations

of white spruce in the mixed-wood forest based on 10 microsatellite loci.

| FT | Rep | HT | SP | A_T_ | A | A_e_ | A_R_ | A_P_ | H_o_ | H_e_ | *F* |
| --- | --- | --- | --- | --- | --- | --- | --- | --- | --- | --- | --- |
| **(1) Individual subpopulation** | | | | | | | | | | | |
| MW | R1 | CON | E928 | 105 | 10.50 (2.08) | 5.47 (1.30) | 10.06 | 0 | 0.552 (0.082) | 0.652 (0.091) | 0.152 (0.049) |
| MW | R1 | 75R | E912 | 115 | 11.50 (2.57) | 7.32 (1.97) | 10.45 | 1 | 0.583 (0.083) | 0.677 (0.093) | 0.132 (0.060) |
| MW | R1 | 50R | E911 | 115 | 11.50 (2.67) | 6.97 (1.82) | 9.87 | 0 | 0.526 (0.083) | 0.652 (0.103) | 0.166 (0.057) |
| MW | R1 | 20R | E910 | 116 | 11.60 (2.46) | 6.85 (1.68) | 10.38 | 0 | 0.527 (0.080) | 0.663 (0.095) | 0.197 (0.058) |
| MW | R1 | 10R | E913 | 109 | 10.90 (2.42) | 6.82 (1.80) | 9.71 | 1 | 0.440 (0.065) | 0.645 (0.103) | 0.253 (0.064) |
| MW | R1 | CCT | E914 | 119 | 11.90 (2.51) | 7.40 (1.96) | 9.45 | 0 | 0.531 (0.098) | 0.666 (0.099) | 0.183 (0.072) |
| MW | R2 | CON | E902 | 123 | 12.30 (2.78) | 6.95 (1.90) | 10.42 | 2 | 0.521 (0.080) | 0.659 (0.100) | 0.189 (0.053) |
| MW | R2 | 75R | E906 | 116 | 11.60 (2.56) | 7.24 (1.92) | 10.87 | 0 | 0.488 (0.089) | 0.661 (0.092) | 0.283 (0.067) |
| MW | R2 | 50R | E903 | 114 | 11.40 (2.37) | 6.83 (1.63) | 10.93 | 1 | 0.563 (0.087) | 0.649 (0.106) | 0.113 (0.038) |
| MW | R2 | 20R | E905 | 106 | 10.60 (2.40) | 6.63 (1.90) | 10.49 | 0 | 0.518 (0.091) | 0.632 (0.100) | 0.207 (0.064) |
| MW | R2 | CCT | E899 | 114 | 11.40 (2.59) | 6.66 (1.71) | 10.23 | 0 | 0.575 (0.079) | 0.649 (0.096) | 0.071 (0.051) |
| **(2) Means mixed-wood** | | | | | | | | | | | |
|  |  | CON |  | 114.0 | 11.40 | 6.21 | 10.24 | 1.0 | 0.537 | 0.656 | 0.171 |
|  |  | 75R |  | 115.5 | 11.55 | 7.28 | 10.66 | 0.5 | 0.536 | 0.669 | 0.208 |
|  |  | 50R |  | 114.5 | 11.45 | 6.90 | 10.40 | 0.5 | 0.545 | 0.651 | 0.140 |
|  |  | 20R |  | 111.0 | 11.10 | 6.74 | 10.44 | 0.0 | 0.523 | 0.648 | 0.202 |
|  |  | 10R |  | 109.0 | 10.90 | 6.82 | 9.71 | 1.0 | 0.440 | 0.645 | 0.253 |
|  |  | CCT |  | 116.5 | 11.65 | 7.03 | 9.84 | 0.0 | 0.553 | 0.658 | 0.127 |
| **(3) Overall mean MW** | | | | 113.8 | 11.38 | 6.83 | 10.26 | 0.5 | 0.529 | 0.655 | 0.177 |
| **Means over CD and MW** | | | | | | | | | | | |
| Mean CD (from Table 3) | | | | 110.9 | 11.09 | 6.71 | 10.20 | 0.7 | 0.533 | 0.655 | 0.173 |
| Mean MW | | | | 113.8 | 11.38 | 6.83 | 10.30 | 0.5 | 0.529 | 0.655 | 0.177 |

Details of the harvesting treatments (HT) are provided in Table 1. N, number of samples; A_T_, total number of alleles; A, mean number of alleles

per locus; A_e_, effective number of alleles per locus; A_R_, allelic richness; A_P_, private alleles; H_o_, mean observed heterozygosity; H_e_, mean expected

heterozygosity. FT, forest type; Rep, replication; HT, harvesting treatment; SP, sub-population. Values in parentheses are standard errors.

Table S3. Analysis of variance results for different genetic diversity parameters for testing the differences due to harvesting treatments and forest types (Approach 1).

| Parameters | Source | DF | SS | MSS | F value | Pr > F |
| --- | --- | --- | --- | --- | --- | --- |
| *A_T_* | Forest types  Harvesting treatments  Forest types*Harvesting treatments  Error | 1  5  5  11 | 34.610  103.820  22.823  530.000 | 34.610  20.760  4.564  48.180 | 0.72  0.43  0.09 | 0.414  0.818  0.991 |
| *A* | Forest types  Harvesting treatments  Forest types*Harvesting treatments  Error | 1  5  5  11 | 0.346  1.038  0.228  5.300 | 0.346  0.207  0.045  0.480 | 0.72  0.43  0.09 | 0.414  0.818  0.991 |
| *A_e_* | Forest types  Harvesting treatments  Forest types*Harvesting treatments  Error | 1  5  5  11 | 0.083  2.076  0.698  2.500 | 0.083  0.415  0.139  0.227 | 0.37  1.83  0.61 | 0.557  0.188  0.691 |
| *A_R_* | Forest types  Harvesting treatments  Forest types*Harvesting treatments  Error | 1  5  5  11 | 0.005  1.043  1.093  2.420 | 0.005  0.208  0.218  0.220 | 0.02  0.95  0.99 | 0.879  0.488  0.464 |
| *A_P_* | Forest types  Harvesting treatments  Forest types*Harvesting treatments  Error | 1  5  5  11 | 0.346  1.176  2.352  5.500 | 0.346  0.235  0.470  0.500 | 0.69  0.47  0.94 | 0.423  0.790  0.492 |
| *H_o_* | Forest types  Harvesting treatments  Forest types*Harvesting treatments  Error | 1  5  5  11 | 0.0007  0.0073  0.0123  0.0096 | 0.00067  0.00147  0.00240  0.00087 | 0.76  1.69  2.83 | 0.400  0.217  0.069 |
| *H_e_* | Forest types  Harvesting treatments  Forest types*Harvesting treatments  Error | 1  5  5  11 | 0.000007  0.00144  0.00129  0.0025 | 0.000007  0.00028  0.00025  0.00022 | 0.03  1.27  1.14 | 0.869  0.343  0.397 |
| *F* | Forest types  Harvesting treatments  Forest types*Harvesting treatments  Error | 1  5  5  11 | 0.00060  0.0094  0.0123  0.0322 | 0.00060  0.0018  0.0024  0.0029 | 0.20  0.64  0.84 | 0.659  0.672  0.549 |

**Table S4.** Locus-wise mean *F*-statistic estimates

calculated for 23 subpopulations.

| Locus | *F*_IS_ | *F*_IT_ | *F*_ST_ |
| --- | --- | --- | --- |
| RPGSE2 | 0.074 | 0.092 | 0.019 |
| RPGSE5 | 0.268 | 0.283 | 0.020 |
| RPGSE17 | -0.001 | 0.015 | 0.016 |
| RPGSE34 | 0.064 | 0.098 | 0.037 |
| RPGSE35 | 0.416 | 0.473 | 0.098 |
| RPGSE44 | 0.081 | 0.109 | 0.030 |
| SPAG003 | 0.134 | 0.151 | 0.020 |
| PGL14 | 0.170 | 0.191 | 0.026 |
| UAPgGT8 | 0.270 | 0.293 | 0.032 |
| UAPgCA91 | 0.288 | 0.306 | 0.024 |
|  |  |  |  |
| Mean | 0.176 | 0.201 | 0.032 |
| SE | 0.041 | 0.043 | 0.008 |

**Table S5.** Genetic diversity parameters and fixation index (*F*) of unharvested control and post-harvest natural regeneration

subpopulations of white spruce in the conifer-dominated forest based on 6 EST microsatellite loci.

| Forest  Type | Replicate | Harvesting  treatment | | N | A_T_ | | A | | A_e_ | | A_R_ | A_P_ | H_o_ | | H_e_ | *F* | |  |
| --- | --- | --- | --- | --- | --- | --- | --- | --- | --- | --- | --- | --- | --- | --- | --- | --- | --- | --- |
| Individual subpopulation | | | | | | | | | | | | | | | | | | |
| CD | R1 | | CON | 35 | | 37 | | 6.17 | | 2.73 | 5.78 | 0 | | 0.349 | 0.457 | | 0.129 | |
| CD | R1 | | 75R | 30 | | 40 | | 6.67 | | 4.09 | 6.40 | 0 | | 0.385 | 0.458 | | 0.138 | |
| CD | R1 | | 50R | 28 | | 38 | | 6.33 | | 3.99 | 6.15 | 0 | | 0.421 | 0.536 | | 0.196 | |
| CD | R1 | | 20R | 32 | | 38 | | 6.33 | | 3.47 | 6.04 | 0 | | 0.361 | 0.458 | | 0.193 | |
| CD | R1 | | 10R | 25 | | 41 | | 6.83 | | 4.59 | 6.80 | 0 | | 0.467 | 0.523 | | 0.064 | |
| CD | R1 | | CCT | 35 | | 44 | | 7.33 | | 3.53 | 6.60 | 1 | | 0.405 | 0.503 | | 0.159 | |
| CD | R2 | | Con | 35 | | 42 | | 7.00 | | 3.36 | 6.37 | 0 | | 0.415 | 0.465 | | 0.087 | |
| CD | R2 | | 75R | 35 | | 48 | | 8.00 | | 4.46 | 7.33 | 1 | | 0.403 | 0.516 | | 0.237 | |
| CD | R2 | | 50R | 35 | | 39 | | 6.50 | | 3.72 | 6.11 | 1 | | 0.451 | 0.510 | | 0.124 | |
| CD | R2 | | 20R | 35 | | 37 | | 6.17 | | 2.74 | 5.74 | 0 | | 0.401 | 0.476 | | 0.102 | |
| CD | R2 | | 10R | 35 | | 39 | | 6.50 | | 3.15 | 6.00 | 0 | | 0.404 | 0.461 | | 0.227 | |
| CD | R2 | | CCT | 35 | | 42 | | 7.00 | | 2.95 | 6.39 | 1 | | 0.419 | 0.509 | | 0.191 | |
| Mean conifer-dominated | | | | | | | | | | | | | | | | | | |
|  |  | | CON | 35.0 | | 39.5 | | 6.59 | | 3.05 | 6.08 | 0.00 | | 0.382 | 0.461 | | 0.108 | |
|  |  | | 75R | 32.5 | | 44.0 | | 7.34 | | 4.28 | 6.87 | 0.50 | | 0.394 | 0.487 | | 0.188 | |
|  |  | | 50R | 31.5 | | 38.5 | | 6.42 | | 3.86 | 6.13 | 0.50 | | 0.436 | 0.523 | | 0.160 | |
|  |  | | 20R | 33.5 | | 37.5 | | 6.25 | | 3.11 | 5.89 | 0.00 | | 0.381 | 0.467 | | 0.148 | |
|  |  | | 10R | 30.0 | | 40.0 | | 6.67 | | 3.87 | 6.40 | 0.00 | | 0.436 | 0.492 | | 0.146 | |
|  |  | | CCT | 35.0 | | 43.0 | | 7.17 | | 3.24 | 6.50 | 1.00 | | 0.412 | 0.506 | | 0.175 | |

Details of the harvesting treatments are provided in Table 1. A_T_, total number of alleles; A, mean number of alleles per locus;

A_e_, effective number of alleles per locus; A_P_, private alleles; A_R_, allelic richness; H_o_, mean observed heterozygosity;

H_e_, mean expected heterozygosity; *F,* fixation index.

**Table S6.** Genetic diversity parameters and fixation index (*F*) of unharvested control and post-harvest natural regeneration

subpopulations of white spruce in the mixed-wood forest based on 6 EST microsatellite loci.

| Forest  Type | Replicate | Harvesting  treatment | N | A_T_ | A | A_e_ | A_R_ | A_P_ | H_o_ | H_e_ | *F* |
| --- | --- | --- | --- | --- | --- | --- | --- | --- | --- | --- | --- |
| Individual subpopulation | | | | | | | | | | | |
| MW | R1 | Con | 35 | 41 | 6.83 | 2.80 | 6.18 | 0 | 0.461 | 0.494 | 0.106 |
| MW | R1 | 75R | 35 | 44 | 7.33 | 4.11 | 6.83 | 0 | 0.518 | 0.521 | 0.052 |
| MW | R1 | 50R | 35 | 43 | 7.17 | 3.57 | 6.44 | 0 | 0.452 | 0.480 | 0.078 |
| MW | R1 | 20R | 35 | 40 | 6.67 | 3.29 | 6.04 | 0 | 0.389 | 0.494 | 0.196 |
| MW | R1 | 10R | 35 | 36 | 6.00 | 3.38 | 5.57 | 0 | 0.344 | 0.469 | 0.184 |
| MW | R1 | CCT | 35 | 44 | 7.33 | 4.38 | 7.00 | 0 | 0.410 | 0.503 | 0.162 |
| MW | R2 | Con | 35 | 45 | 7.50 | 3.28 | 6.75 | 1 | 0.393 | 0.493 | 0.167 |
| MW | R2 | 75R | 35 | 41 | 6.83 | 3.61 | 6.37 | 0 | 0.340 | 0.492 | 0.323 |
| MW | R2 | 50R | 35 | 42 | 7.00 | 3.74 | 6.48 | 0 | 0.448 | 0.476 | 0.061 |
| MW | R2 | 20R | 35 | 38 | 6.33 | 3.11 | 5.87 | 0 | 0.394 | 0.450 | 0.198 |
| MW | R2 | CCT | 35 | 43 | 7.17 | 3.41 | 6.40 | 0 | 0.444 | 0.474 | 0.017 |
| **Mean mixed-wood** | | | | | | | | | | | |
|  |  | CON | 35 | 43.0 | 7.17 | 3.04 | 6.47 | 0.50 | 0.427 | 0.494 | 0.137 |
|  |  | 75R | 35 | 42.5 | 7.08 | 3.86 | 6.60 | 0.00 | 0.429 | 0.507 | 0.188 |
|  |  | 50R | 35 | 42.5 | 7.09 | 3.66 | 6.46 | 0.00 | 0.450 | 0.478 | 0.070 |
|  |  | 20R | 35 | 39.0 | 6.50 | 3.20 | 5.96 | 0.00 | 0.392 | 0.472 | 0.197 |
|  |  | 10R | 35 | 36.0 | 6.00 | 3.38 | 5.57 | 0.00 | 0.344 | 0.469 | 0.184 |
|  |  | CCT | 35 | 43.5 | 7.25 | 3.90 | 6.70 | 0.00 | 0.427 | 0.489 | 0.090 |

Details of the harvesting treatments are provided in Table 1. A_T_, total number of alleles; A, mean number of alleles per locus;

A_e_, effective number of alleles per locus; A_P_, private alleles; A_R_, allelic richness; H_o_, mean observed heterozygosity; H_e_, mean expected

heterozygosity; *F,* fixation index*.*

**Table S7.** Overall means for genetic diversity parameters and fixation index for unharvested control and post-harvest

natural regeneration of white spruce in the conifer-dominated and mixed-wood forests based on 6 EST microsatellite

loci. Means followed by the same letter are not significantly different according to Duncan’s Multiple Range Test.

| Harvesting  Treatment | N | A_T_ | A | A_e_ | A_R_ | A_P_ | H_o_ | H_e_ | *F* |
| --- | --- | --- | --- | --- | --- | --- | --- | --- | --- |
| CON | 35 | 41.3A | 6.88A | 3.04B | 6.27A | 0.25A | 0.404A | 0.477A | 0.122A |
| 75R | 34 | 43.3A | 7.21A | 4.07A | 6.27A | 0.25A | 0.411A | 0.497A | 0.188A |
| 50R | 33 | 40.5A | 6.75A | 3.76AB | 6.29A | 0.25A | 0.443A | 0.501A | 0.115A |
| 20R | 34 | 38.3A | 6.38A | 3.15B | 5.92A | 0.00A | 0.386A | 0.469A | 0.172A |
| 10R | 32 | 38.7A | 6.44A | 3.71AB | 6.12A | 0.00A | 0.405A | 0.484A | 0.158A |
| CCT | 35 | 43.3A | 7.21A | 3.57AB | 6.60A | 0.50A | 0.419A | 0.497A | 0.132A |

Details of the harvesting treatments are provided in Table 1. A_T_, total number of alleles; A, mean number of alleles per

locus; A_e_, effective number of alleles per locus; A_P_, private alleles; A_R_, allelic richness; H_o_, mean observed heterozygosity; H_e_, mean

expected heterozygosity; *F,* fixation index*.*

**Table S8.** Mean *F*-statistic estimates from six EST microsatellite loci, calculated over all subpopulations and separately by harvesting treatments.

| Harvesting treatment | Number of stands | *F*_IS_ | *F*_IT_ | *F*_ST_ |
| --- | --- | --- | --- | --- |
| All subpopulations | 23 | 0.148 | 0.178 | 0.037 |
| CON | 4 | 0.122 | 0.142 | 0.030 |
| 75R | 4 | 0.188 | 0.206 | 0.022 |
| 50R | 4 | 0.115 | 0.143 | 0.031 |
| 20R | 4 | 0.172 | 0.203 | 0.030 |
| 10R | 3 | 0.158 | 0.178 | 0.024 |
| CCT | 4 | 0.132 | 0.163 | 0.031 |

Details of the harvesting treatments are provided in Table 1.

**Table S9.** Mean and (range) of genetic distances (Nei 1978) among (below the diagonal) and within (on the diagonal) harvesting treatments

based on six EST microsatellite loci.

| Harvesting  treatment | No. of  subpopulations | CON | 75R | 50R | 20R | 10R | CCT |
| --- | --- | --- | --- | --- | --- | --- | --- |
| CON | 4 | 0.025  (0.011-0.044) |  |  |  |  |  |
| 75R | 4 | 0.028  (0.007-0.063) | 0.020  (0.007-0.033) |  |  |  |  |
| 50R | 4 | 0.043  (0.017-0.115) | 0.024  (0.007-0.069) | 0.037  (0.020-0.058) |  |  |  |
| 20R | 4 | 0.028  (0.017-0.068) | 0.037  (0.000-0.110) | 0.058  (0.014-0.167) | 0.035  (0.012-0.053) |  |  |
| 10R | 3 | 0.026  (0.014-0.044) | 0.018  (0.002-0.058) | 0.032  (0.000-0.095) | 0.034  (0.010-0.084) | 0.028  (0.015-0.040) |  |
| CCT | 4 | 0.028  (0.011-0.067) | 0.029  (0.008-0.072) | 0.042  (0.010-0.103) | 0.040  (0.006-0.130) | 0.031  (0.006-0.096) | 0.044  (0.009-0.074) |

Details of the harvesting treatments are provided in Table 1.

**Table S10.** Genetic diversity parameters and fixation index for unharvested control and post-harvest natural regeneration

of white spruce in the conifer-dominated forest based on four genomic microsatellite loci.

| Forest  Type | | Replicate | Harvesting  treatment | N | A_T_ | A | A_e_ | A_R_ | A_P_ | H_o_ | H_e_ | *F* |
| --- | --- | --- | --- | --- | --- | --- | --- | --- | --- | --- | --- | --- |
| Individual subpopulation | | | | | | | | | | | | |
| CD | R1 | | CON | 35 | 78 | 19.50 | 12.04 | 17.50 | 1 | 0.664 | 0.907 | 0.265 |
| CD | R1 | | 75R | 30 | 68 | 17.00 | 11.49 | 15.96 | 0 | 0.758 | 0.908 | 0.167 |
| CD | R1 | | 50R | 28 | 63 | 15.75 | 11.36 | 15.22 | 0 | 0.737 | 0.898 | 0.182 |
| CD | R1 | | 20R | 32 | 72 | 18.00 | 9.95 | 16.33 | 0 | 0.765 | 0.886 | 0.144 |
| CD | R1 | | 10R | 25 | 69 | 17.25 | 12.31 | 17.15 | 1 | 0.733 | 0.914 | 0.199 |
| CD | R1 | | CCT | 35 | 75 | 18.75 | 12.06 | 16.86 | 1 | 0.750 | 0.912 | 0.177 |
| CD | R2 | | Con | 35 | 67 | 16.75 | 11.27 | 15.38 | 0 | 0.650 | 0.905 | 0.280 |
| CD | R2 | | 75R | 35 | 73 | 18.25 | 11.41 | 16.40 | 0 | 0.705 | 0.909 | 0.224 |
| CD | R2 | | 50R | 35 | 77 | 19.25 | 11.43 | 17.22 | 0 | 0.814 | 0.904 | 0.101 |
| CD | R2 | | 20R | 35 | 70 | 17.50 | 11.40 | 15.94 | 1 | 0.707 | 0.907 | 0.218 |
| CD | R2 | | 10R | 35 | 70 | 17.50 | 11.66 | 15.95 | 1 | 0.743 | 0.904 | 0.175 |
| CD | R2 | | CCT | 35 | 64 | 16.00 | 10.65 | 14.86 | 0 | 0.643 | 0.898 | 0.281 |
| Mean conifer-dominated | | | | | | | | | | | | |
|  |  | | CON | 35.0 | 72.5 | 18.13 | 11.66 | 16.44 | 0.50 | 0.657 | 0.906 | 0.273 |
|  |  | | 75R | 32.5 | 70.5 | 17.63 | 11.45 | 16.18 | 0.00 | 0.732 | 0.909 | 0.196 |
|  |  | | 50R | 31.5 | 70.0 | 17.50 | 11.40 | 16.22 | 0.00 | 0.776 | 0.901 | 0.142 |
|  |  | | 20R | 33.5 | 71.0 | 17.75 | 10.68 | 16.14 | 0.50 | 0.736 | 0.897 | 0.181 |
|  |  | | 10R | 30.0 | 69.5 | 17.38 | 11.99 | 16.55 | 1.00 | 0.738 | 0.909 | 0.187 |
|  |  | | CCT | 35.0 | 69.5 | 17.38 | 11.36 | 15.86 | 0.50 | 0.697 | 0.905 | 0.229 |

Details of the harvesting treatments are provided in Table 1. A_T_, total number of alleles; A, mean number of alleles per locus;

A_e_, effective number of alleles per locus; A_P_, private alleles; A_R_, allelic richness; H_o_, mean observed heterozygosity; H_e_, mean expected

heterozygosity; *F,* fixation index.

**Table S11.** Genetic diversity parameters and fixation index for unharvested control and post-harvest natural regeneration

of white spruce in the mixed-wood forest based on four genomic microsatellite loci.

| Forest  type | Replicate | Harvesting  treatment | N | A_T_ | A | A_e_ | A_R_ | A_P_ | H_o_ | H_e_ | *F* |
| --- | --- | --- | --- | --- | --- | --- | --- | --- | --- | --- | --- |
| Individual subpopulation | | | | | | | | | | | |
| MW | R1 | Con | 35 | 64 | 16.00 | 9.47 | 14.53 | 0 | 0.689 | 0.889 | 0.221 |
| MW | R1 | 75R | 35 | 71 | 17.75 | 12.12 | 16.18 | 1 | 0.679 | 0.909 | 0.251 |
| MW | R1 | 50R | 35 | 72 | 18.00 | 12.06 | 16.48 | 0 | 0.636 | 0.909 | 0.299 |
| MW | R1 | 20R | 35 | 76 | 19.00 | 12.20 | 17.21 | 0 | 0.734 | 0.916 | 0.198 |
| MW | R1 | 10R | 35 | 73 | 18.25 | 11.97 | 16.56 | 1 | 0.586 | 0.909 | 0.356 |
| MW | R1 | CCT | 35 | 75 | 18.75 | 11.92 | 17.05 | 0 | 0.712 | 0.910 | 0.215 |
| MW | R2 | Con | 35 | 78 | 19.50 | 12.46 | 17.43 | 1 | 0.712 | 0.909 | 0.217 |
| MW | R2 | 75R | 35 | 75 | 18.75 | 12.69 | 17.07 | 0 | 0.710 | 0.916 | 0.224 |
| MW | R2 | 50R | 35 | 72 | 18.00 | 11.47 | 16.46 | 1 | 0.736 | 0.910 | 0.192 |
| MW | R2 | 20R | 35 | 68 | 17.00 | 11.91 | 15.63 | 0 | 0.705 | 0.905 | 0.220 |
| MW | R2 | CCT | 35 | 71 | 17.75 | 11.52 | 16.19 | 0 | 0.771 | 0.910 | 0.152 |
| Mean mixed-wood | | | | | | | | | | |  |
|  |  | CON | 35 | 71.0 | 17.75 | 10.97 | 15.98 | 0.50 | 0.701 | 0.899 | 0.219 |
|  |  | 75R | 35 | 73.0 | 18.25 | 12.41 | 16.63 | 0.50 | 0.695 | 0.913 | 0.238 |
|  |  | 50R | 35 | 72.0 | 18.00 | 11.77 | 16.47 | 0.50 | 0.686 | 0.910 | 0.246 |
|  |  | 20R | 35 | 72.0 | 18.00 | 12.06 | 16.42 | 0.00 | 0.720 | 0.911 | 0.209 |
|  |  | 10R | 35 | 73.0 | 18.25 | 11.97 | 16.56 | 1.00 | 0.586 | 0.909 | 0.356 |
|  |  | CCT | 35 | 73.0 | 18.25 | 11.72 | 16.62 | 0.00 | 0.742 | 0.910 | 0.184 |

Details of the harvesting treatments are provided in Table 1. A_T_, total number of alleles; A, mean number of alleles per locus;

A_e_, effective number of alleles per locus; A_P_, private alleles; A_R_, allelic richness; H_o_, mean observed heterozygosity; H_e_, mean expected

heterozygosity; *F,* fixation index*.*

**Table S12.** Overall means of genetic diversity parameters and fixation index for unharvested control and

post-harvest natural regeneration of white spruce in the conifer-dominated and mixed-wood forests based

on four genomic microsatellite loci.

| Harvesting  treatment | N | A_T_ | A | A_e_ | A_R_ | A_P_ | H_o_ | H_e_ | *F* |
| --- | --- | --- | --- | --- | --- | --- | --- | --- | --- |
| CON | 35 | 71.8 | 17.94 | 11.31 | 16.21 | 0.50 | 0.679 | 0.902 | 0.246 |
| 75R | 34 | 71.8 | 17.94 | 11.93 | 16.40 | 0.25 | 0.713 | 0.911 | 0.216 |
| 50R | 33 | 71.0 | 17.75 | 11.58 | 16.35 | 0.25 | 0.731 | 0.905 | 0.193 |
| 20R | 34 | 71.5 | 17.88 | 11.37 | 16.28 | 0.25 | 0.728 | 0.903 | 0.195 |
| 10R | 32 | 70.7 | 17.67 | 11.98 | 16.55 | 1.00 | 0.687 | 0.909 | 0.243 |
| CCT | 35 | 71.3 | 17.81 | 11.54 | 16.24 | 0.25 | 0.719 | 0.908 | 0.206 |

Details of the harvesting treatments are provided in Table 1. A_T_, total number of alleles; A, mean number of alleles

per locus; A_e_, effective number of alleles per locus; A_P_, private alleles; A_R_, allelic richness;

H_o_, mean observed heterozygosity; H_e_, mean expected heterozygosity; *F,* fixation index*.*

**Table S13.** Mean *F-*statistic estimates from four genomic microsatellite loci, calculated

over all subpopulations and separately by harvesting treatments.

| Harvesting treatment | No of stands | *F*_IS_ | *F*_IT_ | *F*_ST_ |
| --- | --- | --- | --- | --- |
| All subpopulations | 23 | 0.217 | 0.235 | 0.026 |
| CON | 4 | 0.246 | 0.246 | 0.024 |
| 75R | 4 | 0.216 | 0.216 | 0.017 |
| 50R | 4 | 0.193 | 0.193 | 0.019 |
| 20R | 4 | 0.195 | 0.195 | 0.023 |
| 10R | 3 | 0.243 | 0.243 | 0.018 |
| CCT | 4 | 0.206 | 0.206 | 0.021 |

Details of the harvesting treatments are provided in Table 1.

**Table S14.** Mean and (range) of genetic distances (Nei 1978) among (below the diagonal) and within (on the diagonal) harvesting treatments based on four genomic microsatellite loci.

| Harvesting  treatment | No. of  subpopulations | CON | 75R | 50R | 20R | 10R | CCT |
| --- | --- | --- | --- | --- | --- | --- | --- |
| CON | 4 | 0.208  (0.091-0.366) |  |  |  |  |  |
| 75R | 4 | 0.156  (0.063-0.273) | 0.104  (0.019-0.153) |  |  |  |  |
| 50R | 4 | 0.154  (0.000-0.348) | 0.177  (0.012-0.185) | 0.125  (0.038-0.188) |  |  |  |
| 20R | 4 | 0.171  (0.057-0.348) | 0.135  (0.049-0.314) | 0.139  (0.045-0.336) | 0.200  (0.057-0.422) |  |  |
| 10R | 3 | 0.159  (0.097-0.242) | 0.169  (0.005-0.262) | 0.121  (0.021-0.207) | 0.164  (0.000-0.312) | 0.131  (0.088-0.171) |  |
| CCT | 4 | 0.155  (0.043-0.240) | 0.123  (0.055-0.218) | 0.138  (0.023-0.210) | 0.179  (0.045-0.265) | 0.155  (0.000-0.216) | 0.171  (0.144-0.209) |

Details of the harvesting treatments are provided in Table 1.

**Table S15.** Estimation of genetic diversity measures and inbreeding coefficient calculated for 10, 50, and 100 microsatellite markers from the simulated datasets.

| Treatment | Replication | A/N_A_ | H_o_ | H_e_ | *F*_IS_ |
| --- | --- | --- | --- | --- | --- |
| 10 SSRs | R1 | 21.62 | 0.907 | 0.929 | 0.024 |
|  | R2 | 21.74 | 0.905 | 0.930 | 0.027 |
|  | R3 | 21.77 | 0.904 | 0.930 | 0.027 |
|  | R4 | 21.71 | 0.912 | 0.931 | 0.020 |
|  | R5 | 21.82 | 0.904 | 0.929 | 0.027 |
|  | Mean | 21.73 | 0.906 | 0.930 | 0.025 |
| 50 SSRs | R1 | 20.92 | 0.900 | 0.922 | 0.025 |
|  | R2 | 20.98 | 0.900 | 0.922 | 0.024 |
|  | R3 | 21.07 | 0.901 | 0.922 | 0.023 |
|  | R4 | 20.97 | 0.895 | 0.922 | 0.030 |
|  | R5 | 21.03 | 0.902 | 0.922 | 0.022 |
|  | Mean | 20.99 | 0.900 | 0.922 | 0.025 |
| 100 SSR | R1 | 21.17 | 0.900 | 0.923 | 0.025 |
|  | R2 | 21.13 | 0.899 | 0.922 | 0.026 |
|  | R3 | 21.11 | 0.898 | 0.923 | 0.026 |
|  | R4 | 21.24 | 0.898 | 0.924 | 0.027 |
|  | R5 | 21.17 | 0.902 | 0.923 | 0.023 |
|  | Mean | 21.16 | 0.899 | 0.923 | 0.025 |

A/N_A_, number of alleles; H_o_, mean observed heterozygosity; H_e_, mean expected heterozygosity; *F*_IS_*,* inbreeding coefficient*.*

**Table S16.** Analysis of variance results for different genetic diversity parameters for testing the differences due to harvesting treatments and forest types (pre- vs post-harvest) (Approach 2).

| Parameters | Source | DF | SS | MS | F value | Pr > F |
| --- | --- | --- | --- | --- | --- | --- |
| *A_T_* | Forest types  Harvesting treatments  Forest types*Harvesting treatments  Error | 1  4  4  10 | 9.800  51.300  13.700  108.000 | 9.800  12.820  3.430  10.800 | 0.91  1.19  0.32 | 0.363  0.373  0.860 |
| *A* | Forest types  Harvesting treatments  Forest types*Harvesting treatments  Error | 1  4  4  10 | 0.612  3.206  0.856  6.750 | 0.612  0.801  0.214  0.675 | 0.91  1.19  0.32 | 0.363  0.373  0.860 |
| *A_e_* | Forest types  Harvesting treatments  Forest types*Harvesting treatments  Error | 1  4  4  10 | 0.372  2.014  1.939  2.500 | 0.372  0.503  0.484  0.250 | 1.49  2.01  1.94 | 0.250  0.168  0.180 |
| *A_R_* | Forest types  Harvesting treatments  Forest types*Harvesting treatments  Error | 1  4  4  10 | 0.554  2.971  0.702  6.507 | 0.554  0.742  0.175  0.650 | 0.85  1.14  0.27 | 0.377  0.392  0.890 |
| *H_o_* | Forest types  Harvesting treatments  Forest types*Harvesting treatments  Error | 1  4  4  10 | 0.0048  0.0007  0.0088  0.0337 | 0.0048  0.0002  0.0022  0.0033 | 1.42  0.05  0.66 | 0.260  0.993  0.634 |
| *H_e_* | Forest types  Harvesting treatments  Forest types*Harvesting treatments  Error | 1  4  4  10 | 0.00000  0.00009  0.00018  0.00030 | 0.00000  0.00002  0.00005  0.00003 | 0.00  0.81  1.52 | 1.000  0.546  0.270 |
| *F* | Forest types  Harvesting treatments  Forest types*Harvesting treatments  Error | 1  4  4  10 | 0.0057  0.0009  0.0108 | 0.0057  0.0002  0.0027 | 1.34  0.05  0.64 | 0.273  0.993  0.647 |


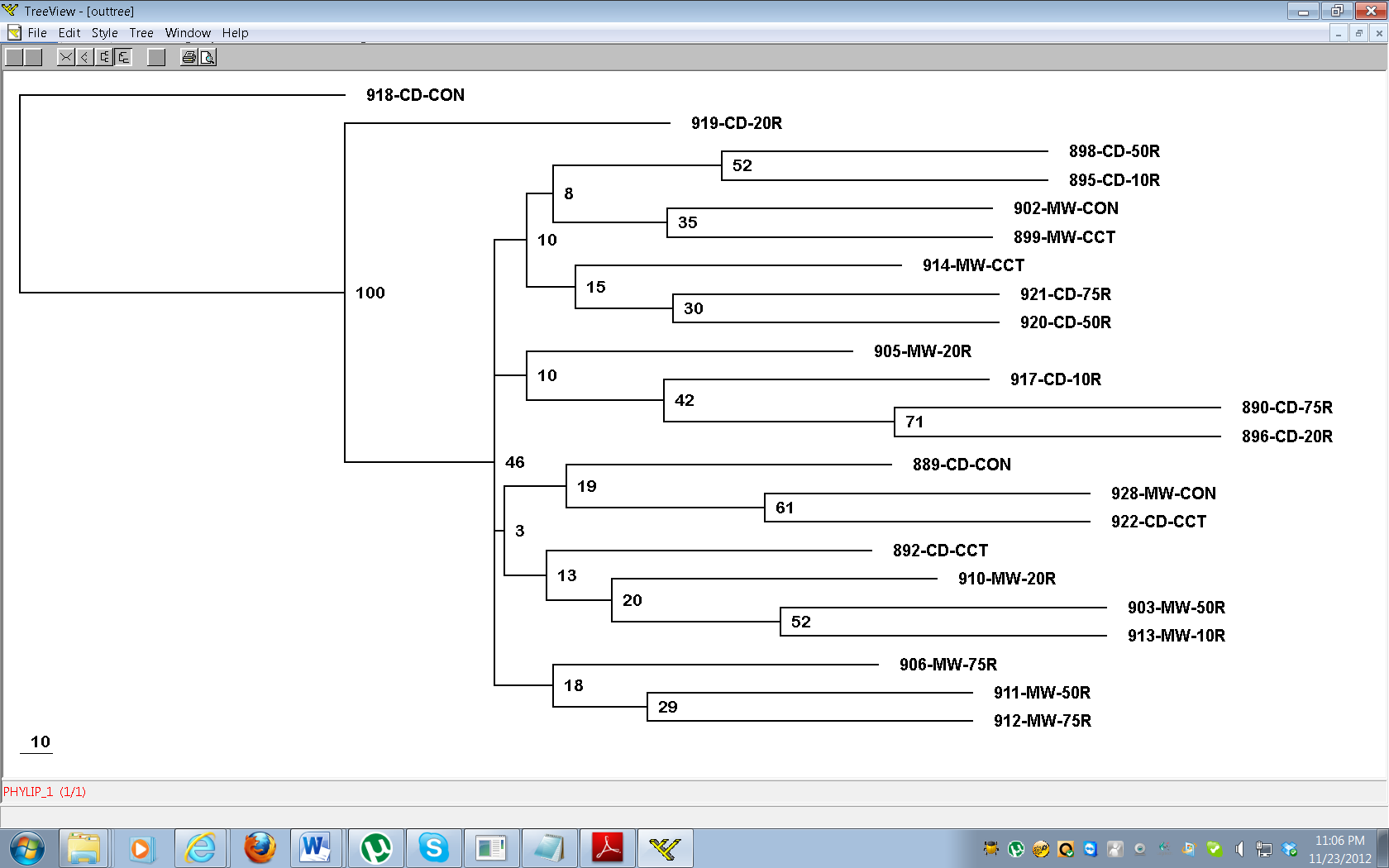


**Figure S1** A neighbor-joining tree, based on Cavalli-Sforza and Edwards’ chord distance *D*_C_ (Cavalli-Sforza and Edwards 1967) from 10 microsatellite loci, showing the relationships among 23 subpopulations of white spruce. CD, conifer-dominated; MW, mixed-wood; CON, unharvested control; 75R, 75% retention; 50R, 50% retention; 20R, 20% retention; 10R, 10% retention; CCT, clearcut.


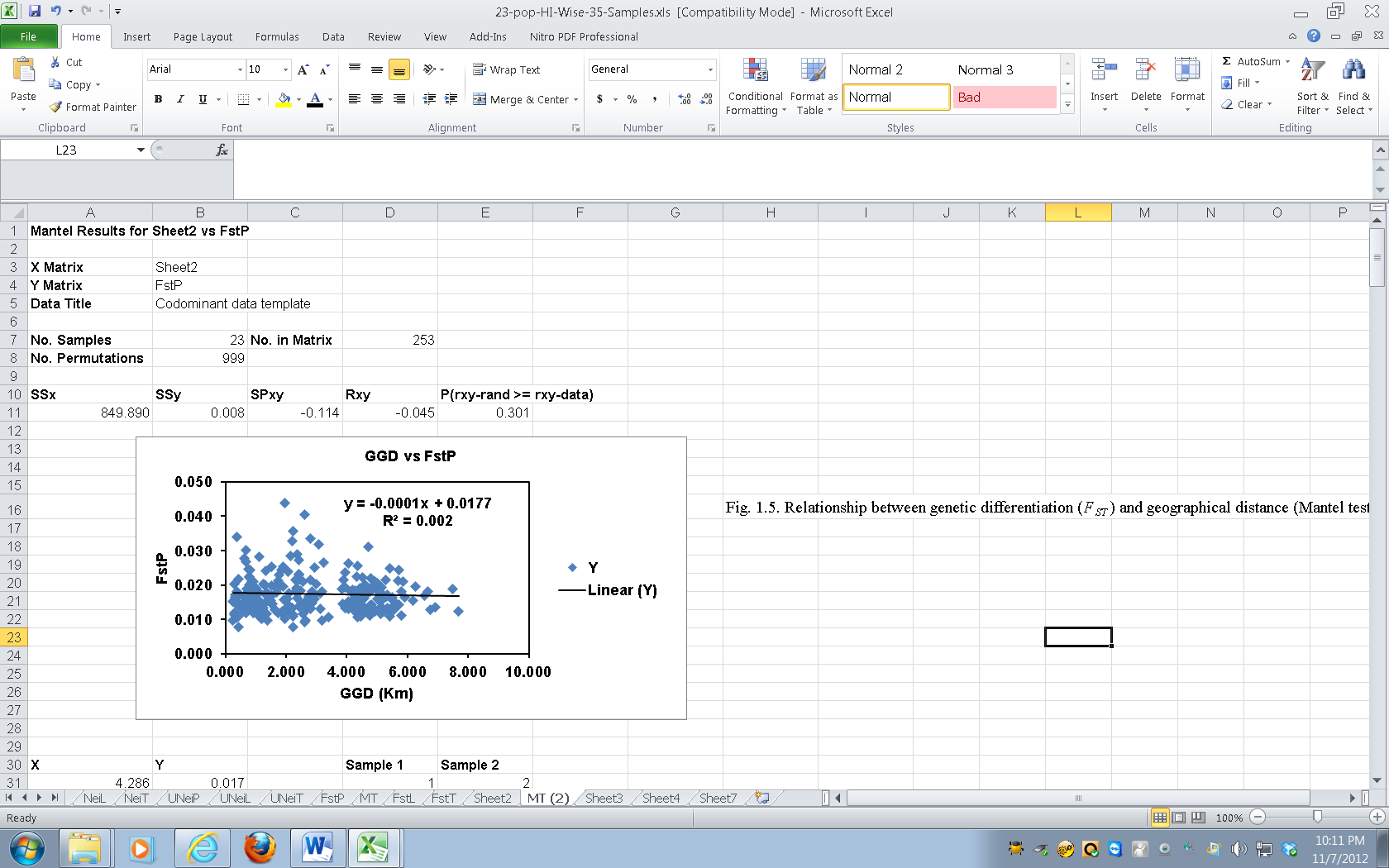


**Figure S2** Relationship between genetic differentiation (*F_ST_*) and geographical distance. No significant isolation by distance (P = 0.301).


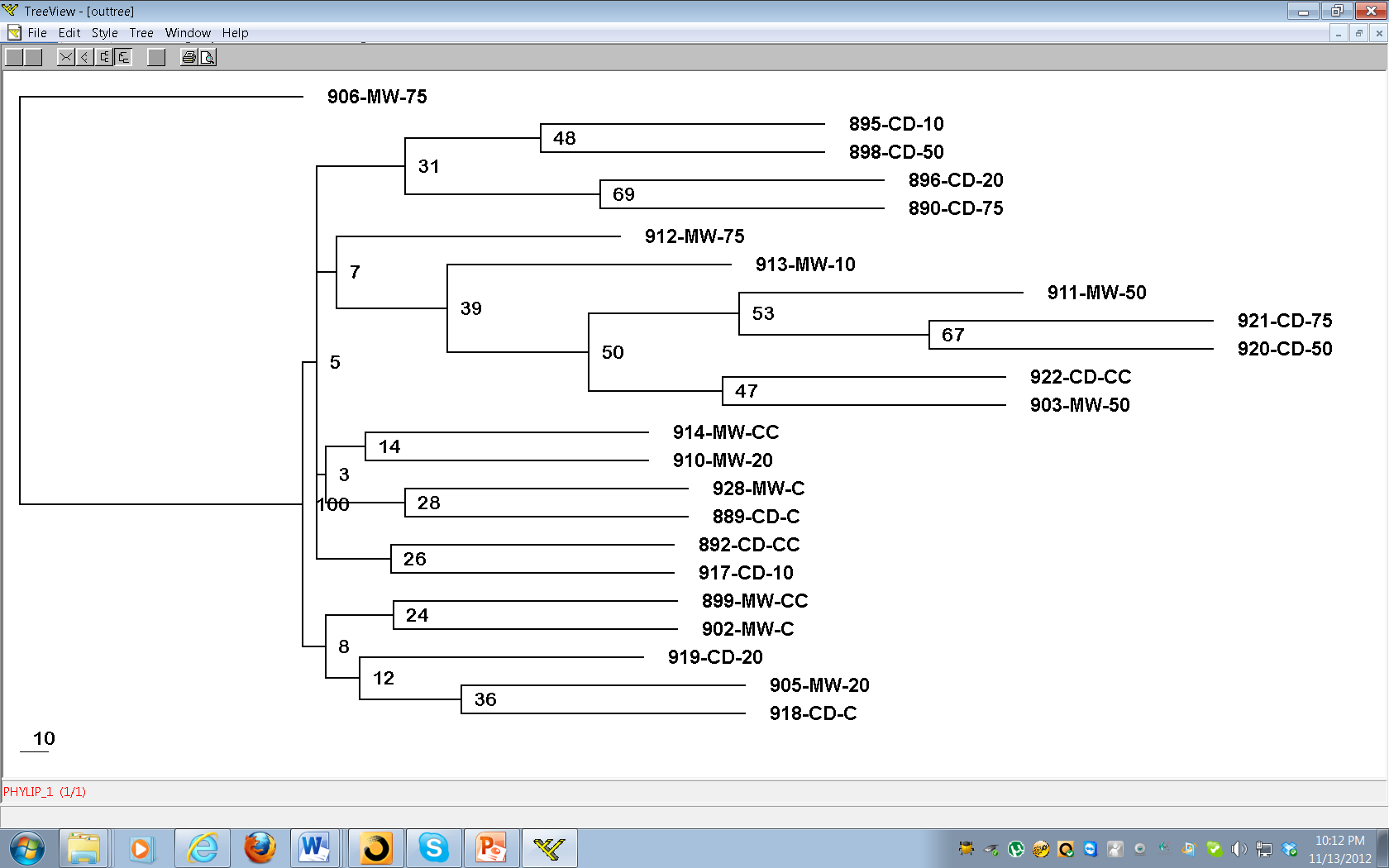


**Fig. S3.** A neighbor-joining tree, based on genetic distances (Nei’s 1978) from six EST-based microsatellite loci, showing the relationships among 23 subpopulations of white spruce. CD, conifer-dominated; MW, mixed-wood; CON, unharvested control; 75R, 75% retention; 50R, 50% retention; 20R, 20% retention; 10R, 10% retention; CCT, clearcut.


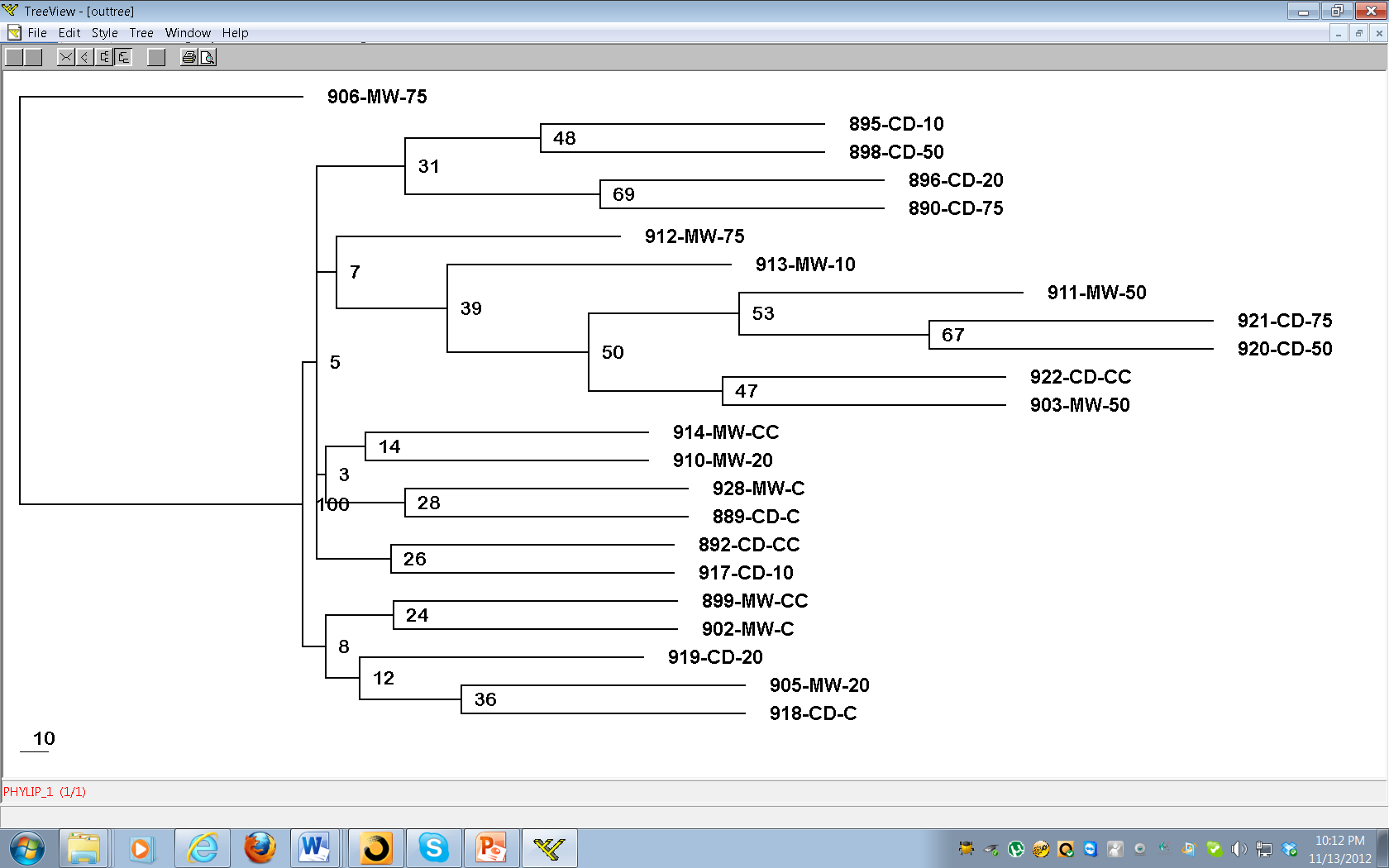


**Figure S4** A neighbor-joining tree, based on Cavalli-Sforza and Edwards’ chord distance *D*_C_ (Cavalli-Sforza and Edwards 1967) for 6 EST microsatellite loci, showing the relationships among 23 subpopulations of white spruce. CD, conifer-dominated; MW, mixed-wood; CON, unharvested control; 75R, 75% retention; 50R, 50% retention; 20R, 20% retention; 10R, 10% retention; CCT, clearcut.


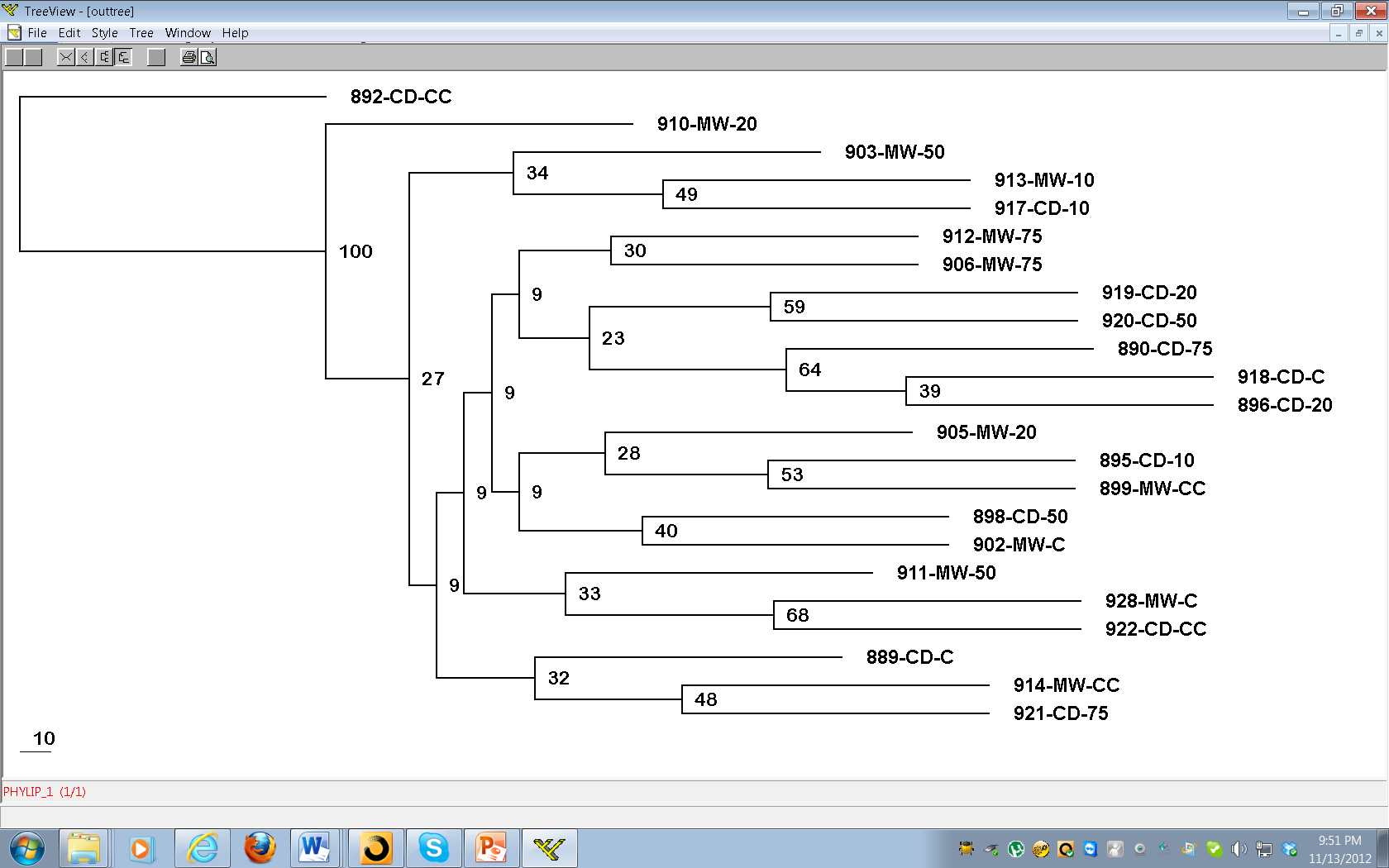


**Figure S5** A neighbor joining tree, based on genetic distances (Nei’s 1978) from four genomic microsatellite loci, showing the relationships among 23 subpopulations of white spruce. CD, conifer-dominated; MW, mixed-wood; CON, unharvested control; 75R, 75% retention;

50R, 50% retention; 20R, 20% retention; 10R, 10% retention; CCT, clearcut.


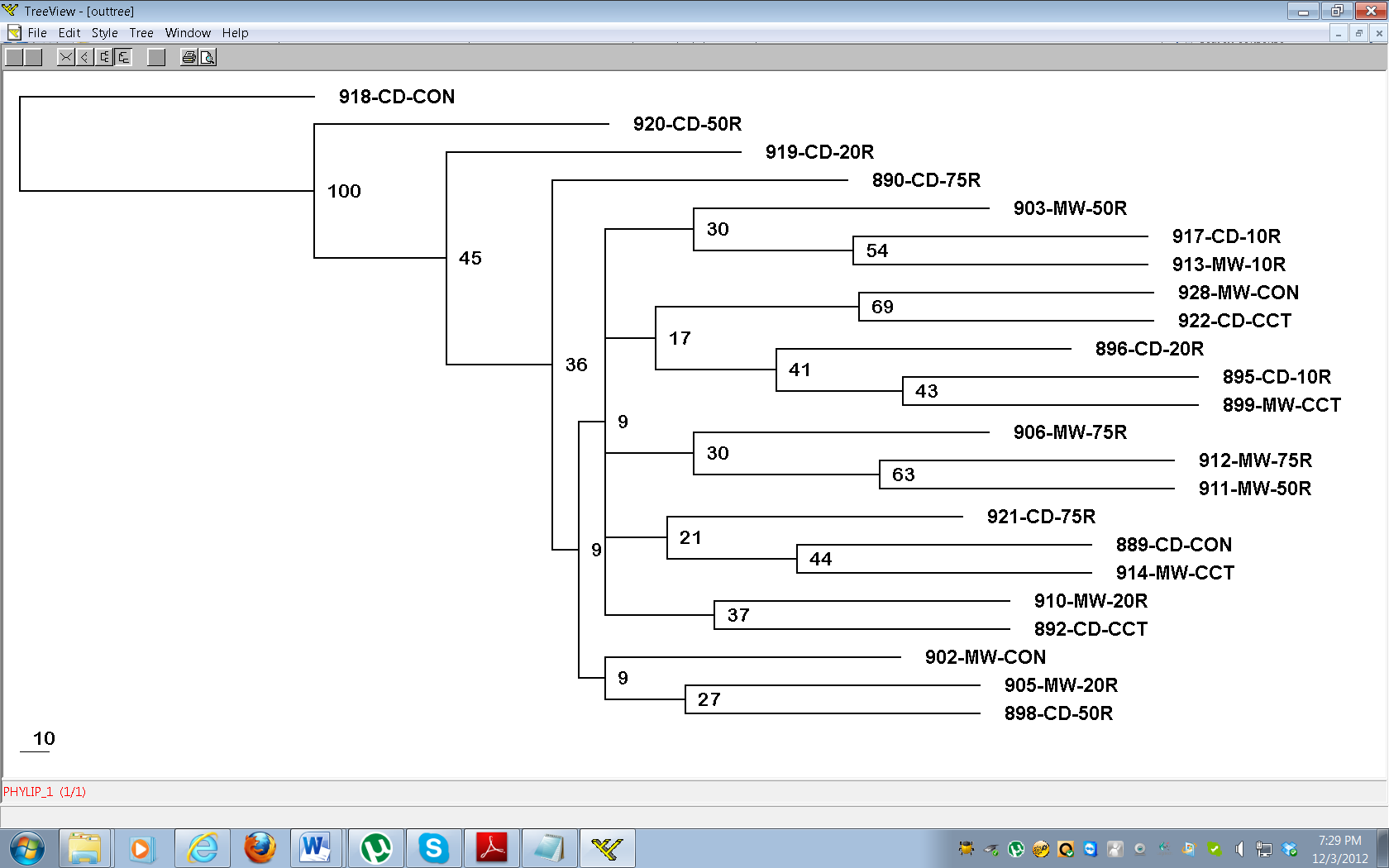


**Figure S6** A neighbor-joining tree, based on Cavalli-Sforza and Edwards’ chord distance *D*_C_ (Cavalli-Sforza and Edwards 1967) for 4 genomic microsatellite loci, showing the relationships among 23 subpopulations of white spruce. CD, conifer-dominated; MW, mixed-wood; CON, unharvested control; 75R, 75% retention; 50R, 50% retention; 20R, 20% retention; 10R, 10% retention; CCT, clearcut.


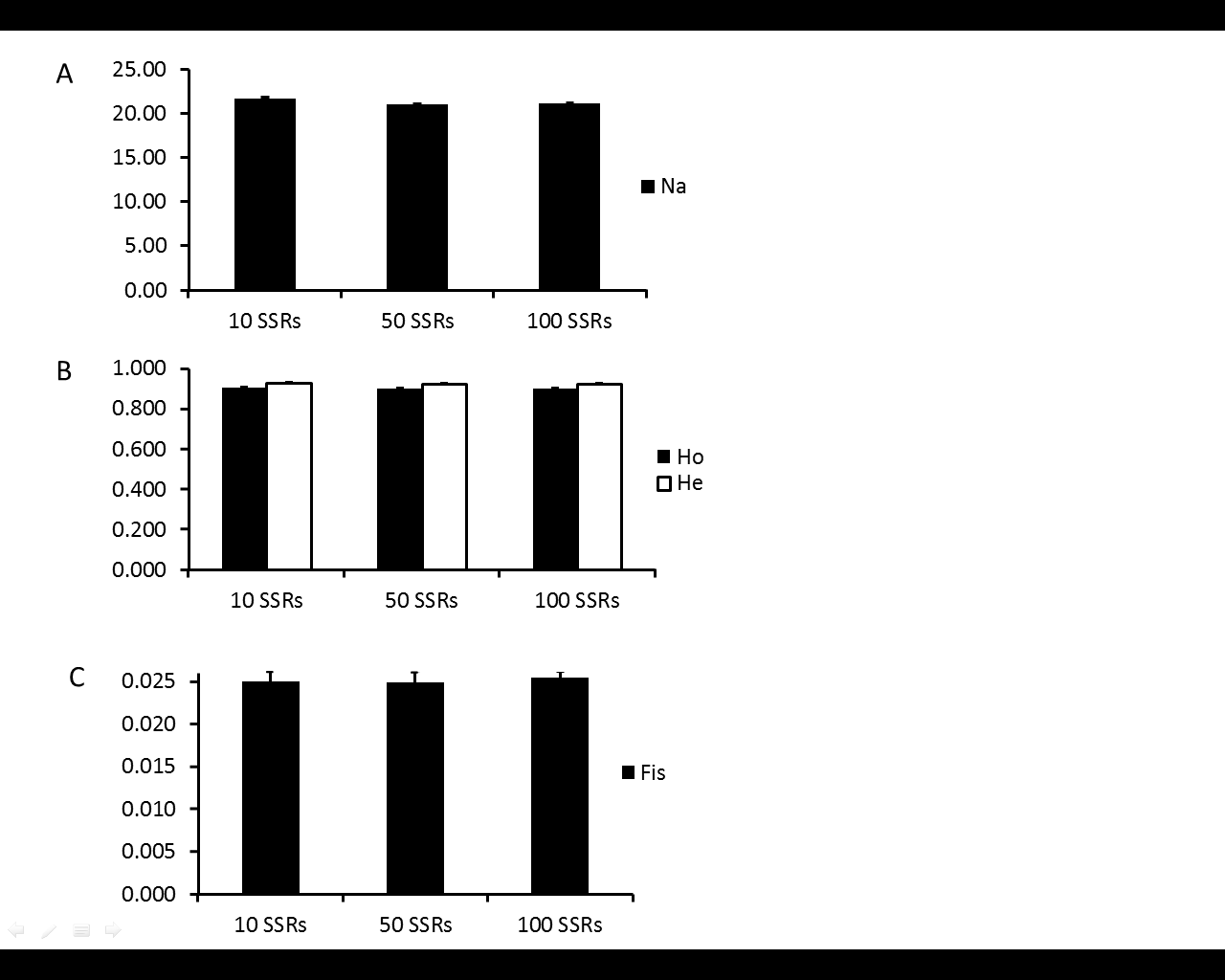


**Figure S7** Estimation of genetic diversity measures and inbreeding coefficient for different number of SSRs (10, 50, and 100) over 20000 generations. In (A): number of alleles (Na), in (B): observed (Ho) and expected heterozygosity (He) and in (C): inbreeding coefficient (Fis).
